# Reagent-Free Prediction of Free-Ammonia Toxicity in Algal Systems Using Chlorophyll Fluorescence Transients and Interpretable Sparse Regression

**DOI:** 10.1021/acs.est.5c18003

**Published:** 2026-06-15

**Authors:** Masatoshi Kishi, Panagiota Karachaliou, Tetsuichi Fujiki, Maki Noguchi Aita, Raúl Muñoz

**Affiliations:** † Institute of Sustainable Processes, 16782 University of Valladolid, Valladolid 47011, Spain; ‡ Department of Chemical Engineering and Environmental Technology, School of Industrial Engineering, University of Valladolid, Dr. Mergelina s/n, Valladolid 47011, Spain; § Research Institute for Global Change, Japan Agency for Marine-Earth Science and Technology, 2-15 Natsushima-cho, Yokosuka, Kanagawa 237-0061, Japan; ∥ Department of Chemical Engineering, 121991 Cyprus University of Technology, 57 Anexartisias Str., Limassol 3603, Cyprus

**Keywords:** biosensor, chlorophyll fluorescence, environmental risk monitoring, free ammonia, microalgae, sparse Lasso regression, wastewater

## Abstract

Accurate assessment of free ammonia (NH_3_) is critical for managing nutrient-rich effluents and algal-based treatment systems, but conventional assays are reagent-intensive and poorly suited to real-time monitoring. Chlorophyll *a* fluorescence offers a rapid, reagent-free proxy for photosynthetic health, yet widely used indices such as *F*_v_/*F*_m_ respond nonspecifically to diverse stresses and rarely yield quantitative NH_3_ concentrations. Here, we develop a reagent-free framework that predicts NH_3_ toxicity in microalgae from fast chlorophyll fluorescence transients (O–J–I–P; OJIP) analyzed with interpretable sparse regression. Three species (*Chlorella vulgaris*, *Acutodesmus obliquus*, and *Arthrospira platensis*) were exposed to environmentally relevant NH_3_ levels, and OJIP responses were monitored for 35 h. Among several multivariate approaches, Lasso regression optimized by nested cross-validation provided the best compromise between accuracy (*R* ^2^ = 0.93–0.98 within species) and transferability across taxa (*R* ^2^ = 0.81–0.87 for green algae). The selected OJIP parameters captured conserved fluorescence signatures of NH_3_-induced stress and retained predictive power under light-induced photoinhibition that markedly depressed *F*_v_/*F*_m_. Incorporating model evaluation diagnostics enabled transparent uncertainty quantification and discrimination of extrapolative predictions. This workflow converts routine OJIP measurements into real-time, reagent-free, and quantitative information on ammonia toxicity in algal cultures and high-ammonia effluents, providing a transparent biosensing platform for environmental risk assessment.

## Graphical abstract

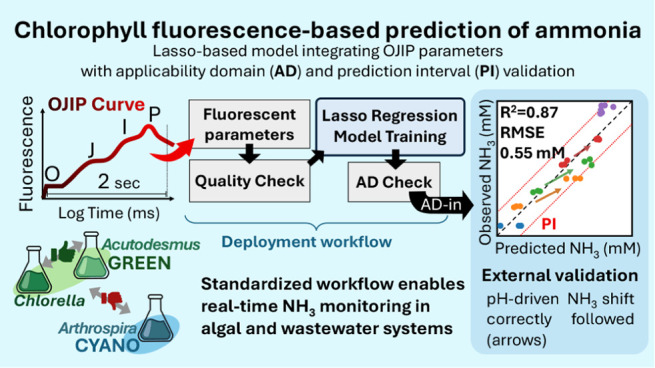

## 1 Introduction

Ammonia (NH_3_/NH_4_^+^) is a major nitrogen species of global environmental concern. Excessive ammonia contributes to eutrophication, oxygen depletion, and toxicity in aquatic ecosystems.^1^ Anthropogenic ammonia emissions have increased sharply over recent decades, driven mainly by fertilizer application and livestock production. For example, Liu et al.^2^ reported that agricultural ammonia emissions increased by 78% between 1980 and 2018. Beaudor et al.^3^ also noted that ∼85% of global anthropogenic ammonia emissions originate from agriculture, particularly fertilizer use and manure management. Together, these trends emphasize the growing ecological and toxicological importance of ammonia pollution.

In industrial and livestock wastewaters, ammonia often occurs at particularly high levels. Thus, total ammoniacal nitrogen (TAN; the sum of NH_3_ and NH_4_^+^) frequently exceeds 200 mg-N L^–1^ (>14 mM) in anaerobic digestion effluents, and in high-strength digestates from livestock or food waste, concentrations can reach several grams per liter. For instance, in food-waste anaerobic digestate, NH_4_^+^-N concentrations of up to 2.6 g L^–1^ (>180 mM) have been reported,^4^ while another study documented TAN levels reaching 3.1 g-N L^–1^ (>220 mM) under mesophilic conditions.^5^ Although TAN comprises both ammonium (NH_4_^+^) and free ammonia (NH_3_), toxicity arises mainly from the unionized free form, which readily diffuses across membranes and disrupts proton gradients.^6^ The fraction of TAN present as NH_3_ increases with pH, so that in alkaline digestates, free ammonia may accumulate to concentrations exceeding the tolerance thresholds of aquatic organisms and microalgae.

Such high NH_3_ concentration poses a major challenge to algae-based systems^7,8^ and also represents a key hazard for aquatic environments, because most phytoplankton species exhibit limited tolerance to NH_3_ toxicity. For phototrophic organisms, free ammonia tolerance thresholds are relatively low: IC_50_ values of 27–44 mg-NH_3_ L^–1^ (22–36 mg-N L^–1^; 1.6–2.6 mM) have been reported for several algal and cyanobacterial species.^9^ Indeed, when cultivating microalgae on anaerobic digestate, a 5- to 10-fold dilution is commonly required to mitigate ammonia toxicity.^10,11^ Such overlaps between environmental ammonia concentrations and biological sensitivity highlight the need for improved monitoring of ammonia toxicity in algal cultivation and wastewater treatment systems.

Conventional free ammonia assays—such as colorimetric methods, ion-selective electrodes, and spectrophotometry—are well established but have drawbacks: they require reagents, frequent calibrations, and costly instruments, and they are poorly suited to real-time monitoring in dynamic environments. These limitations have stimulated interest in reagent-free, rapid, and nondestructive sensing approaches capable of directly reflecting biological stress.

Chlorophyll *a* fluorescence provides a noninvasive optical tool for assessing photosynthetic stress, offering a sensitive and reagent-free method for monitoring environmental toxicity. The O–J–I–P (OJIP) transient is the fast chlorophyll *a* fluorescence induction curve from O (*F*_0_) to P (*F*_m_), with intermediate steps such as J (∼2 ms) and I (∼30 ms), sensitively reflecting sequential redox reactions in photosystem II (PSII), capturing changes in electron transport, charge separation, and energy dissipation.^12^ These OJIP phases have been extensively studied as diagnostic indicators of PSII integrity under environmental stress, including exposure to metals, excess light, temperature shifts, and nutrient limitation.^13–15^ Building on this mechanistic basis, fluorescence-based responses have also been utilized in practical biosensing systems. For example, a paper-based algal biosensor employing immobilized *Chlamydomonas reinhardtii* has successfully detected nanomolar-level herbicide via fluorescence,^16^ demonstrating the feasibility of translating PSII photochemical signals into toxicity detection. Most existing biosensors and modeling studies, however, rely on global yield metrics such as *F*_v_/*F*_m_ or on a small number of composite JIP-test indices (e.g., PI_abs_, CPI, PI_cte_)^17^ that combine multiple partial processes into single scalars. While these indices are highly sensitive, they remain nonspecific: they respond similarly to chemically and physically distinct stressors and largely obscure which functional sites within PSII are perturbed.

Recent work has begun to exploit the richer information in OJIP fingerprints using multivariate statistics and machine-learning approaches,^18–21^ but most existing studies still focus on qualitative toxicity discrimination or compress the transient into a few composite indices, thereby reducing mechanistic resolution. This limitation is particularly important for free ammonia, whose toxicity is thought to involve more than one photosynthetic process rather than a single inhibition site. Because such multiphasic perturbation is expected to affect different regions of the OJIP transient,^22,23^ multivariable analysis of OJIP-resolved descriptors may provide better discriminability than single global indices alone. However, a quantitative framework that converts this richer fluorescence information into operationally useful estimates of the NH_3_ concentration is still lacking. This study addresses that need by developing and validating predictive models that link OJIP-derived fluorescence parameters to free ammonia concentrations in algal monocultures. Specifically, we aim to (i) construct sparse regression models that directly output NH_3_ concentrations (mM) from OJIP parameters under relevant exposure scenarios for bioprocess monitoring in algal cultivation and treatment systems where the dominant phototroph is known, (ii) evaluate the extent to which such models transfer across selected phototrophic taxa and dynamic exposure histories, including conditions of light-induced photoinhibition that strongly affect *F*_v_/*F*_m_, and (iii) incorporate applicability domain (AD) and prediction interval (PI) diagnostics to quantify predictive uncertainty and delineate trustworthy predictions. In this context, the cyanobacterium *Arthrospira* was included not to imply direct mechanistic equivalence with green algae but to test whether the overall fluorescence-to-NH_3_ framework retains predictive utility in an engineering-relevant cyanobacterial system. By jointly analyzing descriptors from different regions of the OJIP transient with sparse regression to select those most associated with specific functional sites, the proposed framework seeks to move chlorophyll-fluorescence-based biosensing beyond nonspecific composite indices toward a practical tool for reagent-free, real-time assessment of ammonia toxicity in algal and wastewater systems.

## 2 Materials and Methods

### 2.1 Test Organisms and Culture Conditions

Three phytoplankton species that frequently appear in wastewater treatment or algal cultivation were selected: two green algae *Chlorella vulgaris* Beijerinck SAG 211–11b and *Acutodesmus obliquus* (Turpin) Hegewald et Hanagata SAG 276–3a, and the cyanobacterium *Arthrospira platensis* (Nordstedt) Gomont SAG 21.99. Hereafter, each species is termed *Chlorella*, *Acutodesmus*, and *Arthrospira*, respectively. *Arthrospira* was included because cyanobacteria are relevant in ammonia-rich phototrophic treatment systems.^24,25^ Its inclusion was meant to test whether the proposed fluorescence-based NH_3_ prediction framework retains its predictive value in a cyanobacterial system. However, cyanobacterial OJIP transients are not assumed to be mechanistically equivalent to those of green algae,^26,27^ and cross-taxon comparisons were interpreted cautiously.

Green algae were cultured in Bold’s Basal Medium with 3-fold nitrogen (3N-BBM) supplemented with sodium bicarbonate and Tris-HCl. For the NH_3_ tolerance test, Mg^2+^ was reduced to 10% of the standard recipe to prevent precipitation; this modification did not affect growth over the experimental period. The alkaliphilic cyanobacterium *Arthrospira* was grown in modified Spirulina-Ogawa-Terui (SOT) medium.^28^ Precultures were maintained in aerated Roux glass flasks at 25 °C under continuous illumination (100 μmol photons m^–2^ s^–1^) and continuous media supply with temperature control in a thermostat water bath (CC1 Compatible Control, Huber, Germany). Continuous illumination was used to maintain a stable photoacclimated physiological state and to avoid diel fluctuations in chlorophyll fluorescence kinetics. The dilution rate was maintained at 0.4–0.6 d^–1^ until inoculation.

### 2.2 Controlled Exposure Experiments (Culture 1)

The 50% effective concentration (EC_50_) to NH_3_ was assumed from preliminary experiments as 1.3, 1.9, and 1.6 mM for *Chlorella*, *Acutodesmus*, and *Arthrospira*, respectively. Exposure levels were set at 0%, 50%, 100%, 200%, and 400% of each species’ EC_50_ (denoted N0–N400) to span subinhibitory to strongly inhibitory conditions. Cells were transferred to NH_3_-free media and equally distributed into 150 mL glass serum bottles (120 mL culture volume, triplicates) so that the initial optical density at 750 nm (OD_750_) becomes approximately 0.05. After 10 min of dark acclimation, NH_4_Cl was added to achieve the desired NH_3_ concentration, calculated from total ammoniacal nitrogen (TAN), pH, temperature, and ionic strength (cf. Section 2.5.1), and fluorescence was measured immediately thereafter. The measurement sequence was arranged so that all bottles experienced approximately the same dark-acclimation period before analysis, thereby standardizing the initial photosynthetic state across treatments. Bottles were incubated in a thermostat bath at 25 °C, under 100 μmol photons m^–2^ s^–1^ from white LEDs with stirring (300 rpm for *Chlorella* and *Acutodesmus*, 100 rpm for *Arthrospira*). Stirring was applied to maintain homogeneous suspensions, reduce settling and self-shading, and ensure consistent optical conditions during fluorescence measurement. Because agitation speed was kept constant within each species across all NH_3_ treatments, any mechanical effect would have acted as a shared background condition rather than as a treatment-specific factor. Liquid samples were taken at 0, 6–7, 12–13, 23, and 35–36 h after 10–15 min dark acclimation, and fluorescence, OD750, pH, and TAN were measured. Dynamic NH_3_ concentration was calculated for each sampling time. The inhibitory effect of Cl^–^ from NH_4_Cl was considered negligible, as the maximum concentration of Cl^–^ was approximately 70 mM, at which *Chlorella vulgaris* and *Acutodesmus obliquus*,^29^ as well as *Arthrospira platensis*,^30^ grew slightly more favorably than at lower concentrations.

### 2.3 External Validation Assays (Culture 2)

To test the external validity of the NH_3_ estimation models, short-duration validation cultures (Culture 2) were performed with higher initial cell density (initial OD_750_ of 0.4 as opposed to 0.05 in Culture 1) and shorter cultivation time (14 h). The NH_3_ concentration levels were further raised (0%, 50%, 150%, 350%, and 650%) for *Acutodesmus* and *Arthrospira* so that the validation data set contains sufficiently strong NH_3_ perturbations. Experimental conditions were otherwise similar to those of Culture 1. These validation data sets were obtained from independent laboratory cultures under controlled conditions and did not involve real wastewater matrices. These data sets were not used in model training and served exclusively for external evaluation.

### 2.4 Analytical and Fluorescence Measurement Protocols

Chlorophyll fluorescence was measured with a portable fluorometer (AquaPen CP-110, PSI, Czechia). Three protocols were applied: maximum quantum yield (*F*_v_/*F*_m_) after dark acclimation, fast fluorescence induction (OJIP) curves recorded for 2 s, and a light curve protocol (LC2) consisting of four actinic light levels from 50 to 1000 μmol photons m^–2^ s^–1^ with 30 s intervals. Blue excitation light at 455 nm was used for the two green algae, while red–orange excitation at 630 nm was applied for the cyanobacterium. The longer excitation wavelength was selected for *Arthrospira* because cyanobacterial fluorescence is strongly influenced by phycobilisome-based light harvesting,^27,31^ and preliminary measurements showed that blue excitation did not produce a sufficiently stable induction signal for this species. Consequently, comparisons involving *Arthrospira* were based on normalized and derived OJIP descriptors rather than on absolute fluorescence amplitudes. The saturation pulse intensity was approximately 600 μmol photons m^–2^ s^–1^ as measured using a spherical quantum sensor (US-SQS/L, Walz, Germany) connected to a light meter (LI-250A, LI-COR, USA). In addition, pH (Accumet AB315, Fisher Scientific, USA), optical density at 750 nm (OD_750_) (UV-2550, Shimadzu, Japan), and total ammoniacal nitrogen (TAN, Nessler method) were measured for each sample.

### 2.5 Derived Variables and Fluorescence Data Preprocessing

#### 2.5.1 Growth Rate, NH_3_, and EC_50_ Calculations

The specific growth rates (μ; d^–1^) of each microalga were calculated with growth data after the lag phase (>6 h). For *Acutodesmus* and *Arthrospira*, since the reduction in growth was observed in 23–35 h, the growth data up to 23 h were used. The growth rate was calculated using eq 1:

1 $$\mu =\frac{\ln X_{2}-\ln X_{1}}{t_{2}-t_{1}}$$

where *X*_*i*_ is the OD_750_ at time *t*_*i*_ (d).

Free ammonia was calculated from total ammoniacal nitrogen (TAN) concentration, pH, temperature, and ionic strength.^32^ The p*K*_a_ value was calculated based on ionic strength and temperature as follows:^33–35^

2 $$pK_{a}=\frac{2835.76}{T}-0.6322+0.001225\times T+\left(0.1552-0.0003142\left(T-273.15\right)\right)I_{f}$$

where *T* is the temperature in kelvin (K), *I*_f_ is the ionic strength (mol L^–1^), which was calculated from the medium composition and NH_4_Cl added. The NH_3_ concentration was calculated as follows:

3 $$\left[{\mathrm{NH}}_{3}\right]=K_{a}\times \frac{\left[\mathrm{TAN}\right]}{10^{-pH}+K_{a}}$$

The NH_3_ concentration was not assumed to remain constant after NH_4_Cl addition. Instead, it was recalculated for each sampling time point using the measured TAN and pH values together with the temperature and ionic strength. This time-resolved NH_3_ value was then used as the response variable for the model construction and evaluation.

The 50% effective concentration (EC_50_) was estimated by fitting a two-parameter Hill equation^32^ according to eq 4:

4 $$\mu_{rel}=\frac{1}{1+{\left(\frac{x}{EC_{50}}\right)}^{n}}$$

where μ_rel_ is the relative specific growth rate normalized to the maximum value in the N0 culture, *x* is the NH_3_ concentration (mM), and *n* is the Hill coefficient.

#### 2.5.2 OJIP Preprocessing and Quality Assurance

OJIP transients represent fast chlorophyll *a* fluorescence induction curves recorded after illumination of dark-adapted photosynthetic samples. The fluorescence intensity rises from the origin (O) to the maximum (P) through distinct inflection points—K (≈300 μs), J (≈2–3 ms), and I (≈30 ms)—that correspond to sequential reductions of electron carriers within photosystem II (PSII), including the oxygen-evolving complex (OEC), the primary quinone acceptor QA, and the plastoquinone (PQ) pool. These characteristic phases provide diagnostic information on the redox dynamics and functionality of the photosynthetic electron transport chain.^12,36–39^

Raw OJIP traces were first resampled onto a logarithmic sequence of time points, regularly spaced along a log-transformed time axis.^40^ The resampled curves were then smoothed using a cubic smoothing spline (scipy.interpolate.UnivariateSpline), with smoothing factors adjusted to preserve the O–K–J–I–P kinetics while reducing high-frequency noise. Smoothed traces were subsequently normalized at O (minimum fluorescence; *F*_0_) and P (maximum fluorescence; *F*_m_), and fluorescence parameters were calculated (Table 1; see Supporting Information Table S1 for the full list of the parameters). Instead of utilizing the full set of JIP-test parameters, the parameter set was preselected based on whether the variables (i) directly described OJIP-shape changes (e.g., relative fluorescence values such as *V*_j_) or (ii) represented physiologically interpretable descriptors of electron-transport behavior that were potentially relevant to NH_3_ stress. Parameter-ablation analysis showed that removing many JIP-test-derived parameters had only minor effects on prediction accuracy, whereas excluding the area-related descriptors (*S*_m_ and *N*) reduced performance more clearly (see Supporting Information Section 1b). After parameter calculation, quality assurance was applied by excluding measurements with abnormal curve shapes, unstable baselines, or insufficient signal-to-noise ratios (see Supporting Information for detailed criteria). In highly inhibited cells during the latter culture period, fluorescence signals were markedly reduced, resulting in a low signal-to-noise ratio (cf. Supporting Information Figure S1). Thus, the quality assurance resulted in the exclusion of most N400 samples (except Time 0 in all species) and most N200 samples in *Arthrospira*. The results on most fluorescence parameters (except for *F*_v_/*F*_m_) presented in this paper are based on the selected samples.

**Table 1:** Principal OJIP-Derived Parameters Discussed in This Work^a^

Parameter	Definition	Formula	Physiological significance
*F*_0_	Minimal fluorescence	*F*_0_	All PSII RCs open
*F*_m_	Maximal fluorescence	*F*_m_	All PSII RCs closed
*F*_v_/*F*_m_	Maximum quantum yield of primary photochemistry	$\frac{F_{v}}{F_{m}}=\frac{F_{m}-F_{0}}{F_{m}}$	PSII efficiency
*F*_p_/*F*_max_	Ratio of P amplitude to max signal^b^	$\frac{F_{p}}{F_{max}}$	Relative elevation of the J region against the terminal fluorescence peak
*V*_k_	Relative variable fluorescence at 300 μs	$\frac{F_{300\mu s}-F_{0}}{F_{m}-F_{0}}$	Early PSII perturbation during the O–J
*V*_j_	Relative variable fluorescence at J step (2 ms)	$\frac{F_{j}-F_{0}}{F_{m}-F_{0}}$	J-step accumulation (QA-related)
*V*_i_	Relative variable fluorescence at I step (30 ms)	$\frac{F_{i}-F_{0}}{F_{m}-F_{0}}$	Later acceptor-side reduction state (PQ-pool-related)
*V*_k_/*V*_j_	Ratio of K to J step	$\frac{F_{300\mu s}-F_{0}}{F_{j}-F_{0}}$	Early donor-side-associated perturbation relative to the J step
*M*_0_	Approximated initial slope of fluorescence rise	$\frac{4\left(F_{300\mu s}-F_{0}\right)}{F_{m}-F_{0}}$	Initial rate of fluorescence rise (early PSII closure dynamics)
Area	Integrated area above the fluorescence curve up to $t_{F_{m}}$	$$\int_{t_{0}}^{t_{F_{m}}}\left(F_{m}-F\left(t\right)\right)dt$$	Integrated acceptor-side capacity beyond QA
*S*_m_	Normalized area above the fluorescence curve	$\frac{\mathrm{Area}}{F_{m}-F_{0}}$	Size of electron-acceptor pool
*N*	Number of QA reduction events until *F*_m_	$\frac{S_{m}\times M_{0}}{V_{j}}$	Multiturnover electron-acceptor capacity up to *F*_m_

a Only the parameters most critical for interpreting photosynthetic responses are summarized here. A full compilation of parameters is provided in the Supplementary Materials. Parameters are mostly derived from Strasser et al., 2004.^37^

b Custom parameter. Abbreviations: PSII = photosystem II; RC = reaction center; QA = primary quinone electron acceptor of PSII; PQ = plastoquinone pool (secondary electron acceptor beyond QA); and OEC = oxygen-evolving complex.

For LC2 measurements, representative indices including ΦII (effective quantum yield of PSII photochemistry), NPQ (nonphotochemical quenching coefficient, reflecting heat dissipation capacity), and qP (photochemical quenching coefficient, representing the proportion of open PSII centers) were obtained at each actinic light intensity within 50–1000 μmol photons m^–2^ s^–1^.^31^ These indices were used only for comparison with OJIP-derived models.

### 2.6 Model Construction and Statistical Analysis

Growth and fluorescence responses across treatments were compared by one-way ANOVA followed by Tukey–Kramer post hoc tests.

#### 2.6.1 Multivariable Modeling of NH_3_ Prediction

Recent advances in machine learning have increasingly enabled the quantitative estimation of environmental indicators from complex sensor signals.^41^ Chlorophyll *a* fluorescence is particularly well suited yet underexploited to such data-driven approaches because OJIP transients provide high-dimensional yet mechanistically interpretable fingerprints of photosynthetic status. In this study, we therefore applied multivariable modeling to predict free ammonia (NH_3_) concentrations from chlorophyll fluorescence–derived parameters (hereafter referred to as features in the context of model construction). Three families of multivariable analyses were deployed: Canonical Analysis of Principal Coordinates (CAP), regularized linear regressions (Ridge and Lasso), and tree-based ensemble regressors (Random Forests and Gradient Boosting). The former two model families were first attempted to distinguish the five levels of NH_3_ categorically (N0 to N400) through discrimination analyses. Then, regression analyses were attempted to predict the actual NH_3_ concentrations in the algal cultures. The models constructed with data from *Chlorella* were used for the comparison, as this data set was more robust and represented both light inhibition and recovery more than the other species.

Canonical Analysis of Principal Coordinates (CAP) was included to ensure continuity with fluorescence-based discrimination studies (Duarte et al., 2021). Ridge and Lasso (names derived from “Least Absolute Shrinkage and Selection Operator”) regressions are regularized multiple regression methods that incorporate penalty terms to avoid overfitting and to address the effect of collinearity among variables.^43,44^ While Ridge regression retains all variables with shrunk coefficients, Lasso regression performs variable selection by reducing some coefficients to zero, making it a powerful tool to construct compact and versatile predictive models, which are frequently applied in machine learning.^45,46^ Although intermediate approaches such as the Elastic Net combine both L1 and L2 penalties,^46^ this study focused on comparing Ridge and Lasso as representative extremes of regularization. Tree-based ensemble regressors, including Random Forest and Gradient Boosting, were additionally tested as nonlinear counterparts to the regularized linear models. Random forest constructs an ensemble of decision trees trained on bootstrapped subsets of data and averages their predictions to reduce variance and enhance generalization.^47^ Gradient boosting sequentially builds weak learners that iteratively minimize residual errors through gradient descent in function space, enabling flexible modeling of complex nonlinear relationships.^48^

For the CAP analysis, canonical discrimination analysis (CDA) and canonical correlation analysis (CCorA) were employed for categorical and regression analyses, respectively,^49^ using the PRIMER-e software. The categorization analysis of Ridge and Lasso was conducted after the regression analysis with the Python library Scikit-learn (sklearn.discriminant_analysis.LinearDiscriminantAnalysis).

To compare five modeling approaches (CCorA, Ridge, Lasso, Random Forest, and Gradient Boosting), preprocessed OJIP-derived features from *Chlorella* cultures were used as explanatory variables, and the measured NH_3_ concentration (mM) was used as the response variable. Model hyperparameters were optimized by grouped nested cross-validation (CV) (see Supporting Information Section 6). The resulting optimized models were first evaluated within the *Chlorella* data set and were subsequently applied to *Acutodesmus* and *Arthrospira* data sets to examine cross-species transferability. Model performance was compared using *R* ^2^, Pearson’s *r*, and RMSE.

In this study, Lasso regression was employed as the primary predictive model owing to its ability to perform variable selection and maintain robustness across phytoplankton species and cultures. Lasso is one of the most widely used forms of sparse regression, in which variable selection is achieved by shrinking many coefficients to zero. Model optimization was conducted by using grouped nested CV with repetition.^50–52^ In Lasso analysis, the training objective is defined according to eq 5:

5 $$L\left(\alpha \right)=\left(1/\left(2n\right)\right)\times {∥y-X\times w∥}^{2}+\alpha \times {∥w∥}_{1}$$

where *L* is the objective function (squared error plus L1 penalty); *X* is the fluorescent feature matrix (*n* × *p*); *y* is the target vector (*n* × 1); *w* is the coefficient vector (*p* × 1); *n* is the number of training samples; α is the L1 penalty strength (often written as λ in other conventions, with λ = 2*n* · α under an alternative scaling); the coefficients were estimated by minimizing *L*(α) with respect to *w* at each α. The α chosen in the inner CV loop was then fixed when refitting on each outer-training split and used to predict the corresponding outer-validation fold.

To qualify prediction reliability, we applied two diagnostics: the applicability domain (AD), which delineates the region of fluorescence-feature space represented by the training data, and the prediction interval (PI), which reports the expected uncertainty for each individual estimate. Samples outside the AD were flagged as extrapolations; wide PIs indicated high uncertainty even within the domain. Implementation details (distance-based AD; PI calibration) are provided in the Supporting Information. For external validation, AD thresholds and PI limits were calibrated exclusively from the corresponding training data and then applied unchanged to validation samples; no validation-set recalibration or threshold adjustment was performed. Regression accuracy with true observed values was also evaluated by performance metrics including the coefficient of determination (*R* ^2^), root-mean-square error (RMSE), and Pearson’s correlation coefficient (*r*). Metrics were computed for out-of-fold predictions and, where applicable, external validation. RMSE is reported in mM NH_3_. Details are described in Supporting Information.

In addition to Lasso, Ridge regression and two tree-based ensemble regressors were trained as comparative models. All models were implemented in Python using the scikit-learn library and optimized under the identical grouped nested CV protocol used for Lasso with hyperparameters tuned in the inner folds.

## 3 Results and Discussion

### 3.1 Physiological and Growth Responses to Ammonia Exposure

The growth of all three microalgae was inhibited from low ammonia concentration (N50), and the growth ceased at high ammonia concentrations (N200 and N400) in all cultures (Figure 1A), confirming the acute physiological toxicity of free ammonia. The maximum specific growth rates obtained in assays without ammonia (N0) were 1.47 ± 0.03, 1.71 ± 0.02, and 0.93 ± 0.06 d^–1^ for *Chlorella*, *Acutodesmus*, and *Arthrospira*, respectively. The growth rates of *Chlorella* and *Acutodesmus* were comparable to literature values, suggesting that there was no other strong toxicity other than ammonia in the culture. On the other hand, that of *Arthrospira* showed slightly lower values than those reported in the literature, which suggests partial metabolic damage. The relatively lower growth rate of *Arthrospira* could be due to physical stress from agitation, as a lower growth rate was observed at higher rotation speeds. This potential confound was constant within species across NH_3_ treatments, but it may have increased the baseline physiological sensitivity of *Arthrospira* and is therefore acknowledged as a limitation.

**Figure 1:**
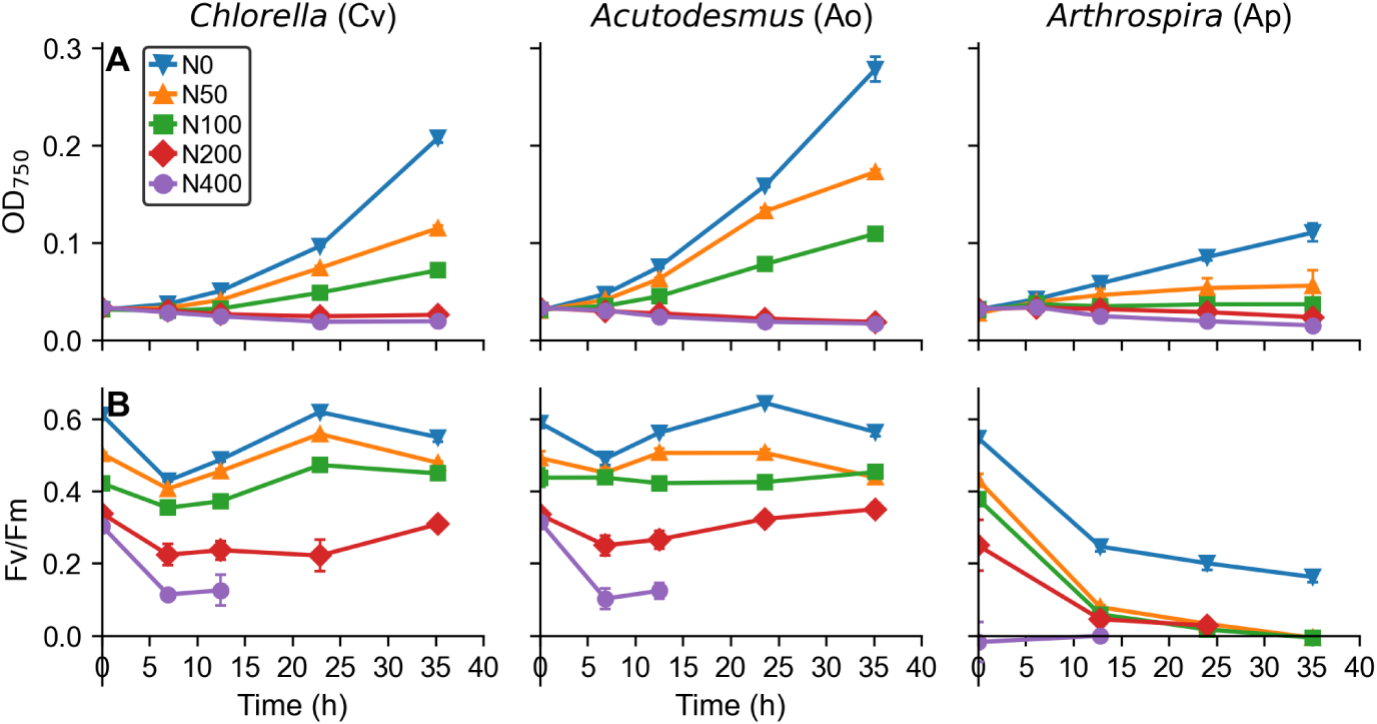
Growth curve and photosynthetic activity of the three species. (A) Growth and (B) maximum quantum yield of PSII (*F*_v_/*F*_m_) over the culture period. Error bars represent standard deviations of triplicate cultures.

The EC_50_ obtained in these culture tests were 1.18, 2.07, and 0.61 mM NH_3_ for *Chlorella*, *Acutodesmus*, and *Arthrospira*, respectively (Figure 2). These values were lower than the literature values of similar species: *Chlorella* 1.6–3.57 mM; *Acutodesmus* (*Scenedesmus*) 3.08 mM; and *Arthrospira* (*Limnospira* and/or *Spirulina*) 2.6–4.9 mM.^9,32,53,54^ These lower EC_50_ values could be due to the very diluted culture (initial OD_750_ of 0.05) with moderate light intensity (100 μmol m^–2^ s^–1^), since light intensity is known to increase the level of toxicity of NH_3_ to algae.^55^ The combined damage by agitation may have further induced vulnerability in *Arthrospira*.

**Figure 2:**
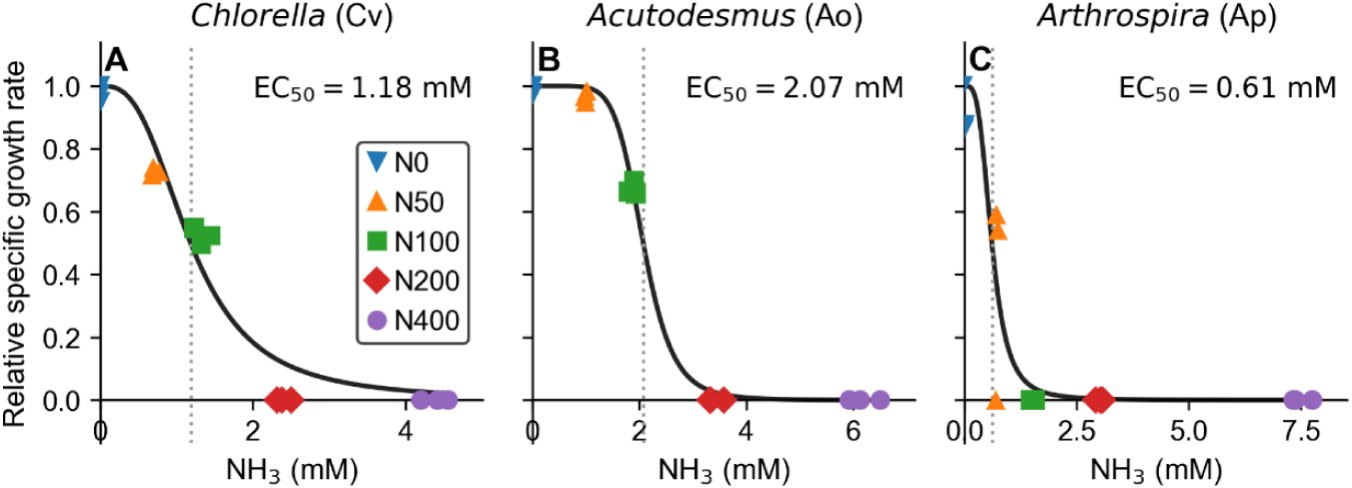
(A-C) Ammonia toxicity characteristics of the three species. The relative specific growth rate was calculated by taking the highest value as the denominator. Negative values were clipped to 0. The 50% effective concentration (EC_50_) values were calculated by fitting a two-parameter Hill equation.

The maximum quantum yield of photosystem II (PSII) was estimated with a fluorescent parameter *F*_v_/*F*_m_ (Figure 1B), which generally shows a good correlation with microalgal and cyanobacterial growth. The initial *F*_v_/*F*_m_ values of the control cultures of *Chlorella* and *Acutodesmus* were slightly lower than the typical range reported for healthy green microalgae (around 0.7 or higher), likely due to mild stress associated with the transfer from dense precultures to dilute experimental cultures. For *Arthrospira*, *F*_v_/*F*_m_ should be regarded as an apparent rather than absolute maximum PSII quantum yield, because dark acclimation alone may not fully oxidize the shared photosynthetic and respiratory electron carriers in cyanobacteria.^27^ Chemical inhibitors such as DCMU can be used in some contexts to approach full QA reduction and PSII reaction center closure, but they were intentionally avoided here to preserve the reagent-free objective of the study. Interestingly, the values of *F*_v_/*F*_m_ quickly responded to the NH_3_ exposure at least in the order of seconds to minutes, as Time 0 samples exhibited good correlations with the NH_3_ concentrations (Figure 1B), even though they were measured only a few tens of seconds after the introduction of ammonia. This rapid response suggests that chlorophyll fluorescence is a good candidate tool to monitor NH_3_ toxicity in an algal suspension, as suggested with other toxins (Choi et al., 2012).

However, reductions in the *F*_v_/*F*_m_ values, even in the control assay, were observed at time 7 h, as compared to those at Time 0. This reduction was probably due to the transition from dense precultures (OD_750_ range of 1–3) to dilute cultures (OD_750_ = 0.05), resulting in photoinhibition at the first hours of incubation. The *F*_v_/*F*_m_ recovered thereafter in *Chlorella* and *Acutodesmus* most likely owing to the acclimation to the light intensity, while it remained low in *Arthrospira*, probably because of continued stress from light and agitation. In this context, the reduction in *F*_v_/*F*_m_ due to light inhibition is a clear example of the challenges in assessing NH_3_ inhibition during the monitoring of algal suspensions. Because the *F*_v_/*F*_m_ parameter is sensitive to a wide range of stressors affecting photosynthetic systems, relying solely on it may result in inaccurate estimations of NH_3_ inhibition (see Supporting Information Section 4 for the correlation). This emphasizes the need to explore photosynthetic parameters that can specifically detect the NH_3_ toxicity.

### 3.2 Fluorescence Responses and Diagnostic Indicators of NH_3_ Toxicity

#### 3.2.1 Fast Fluorescence Induction (OJIP) Curve

There were several visible characteristics of NH_3_-inhibited cells in the OJIP curves. In the raw OJIP curve, an increase in *F*_0_ (O step; minimal fluorescence, when PSII reaction centers are functionally open) was recorded in all three cultures (Figure 3A). Such an increase in *F*_0_ is not unique to NH_3_ and has also been reported under other abiotic stressors (e.g., heat, salinity, and high light).^39,56,57^ Previous research conservatively attributed the increase of *F*_0_ to early PSII perturbation, potentially involving donor-side limitation including the oxygen evolving complex (OEC).^39,58^ It has been proposed that NH_3_ can perturb photosynthesis through more than one process, including effects associated with PSII donor-side function including the OEC and proton-coupled energy conversion.^32,59,60^ In the present study, the rise in *F*_0_ is interpreted as evidence of early PSII perturbation, potentially involving donor-side limitation, rather than as a direct marker of any single inhibition site.

**Figure 3:**
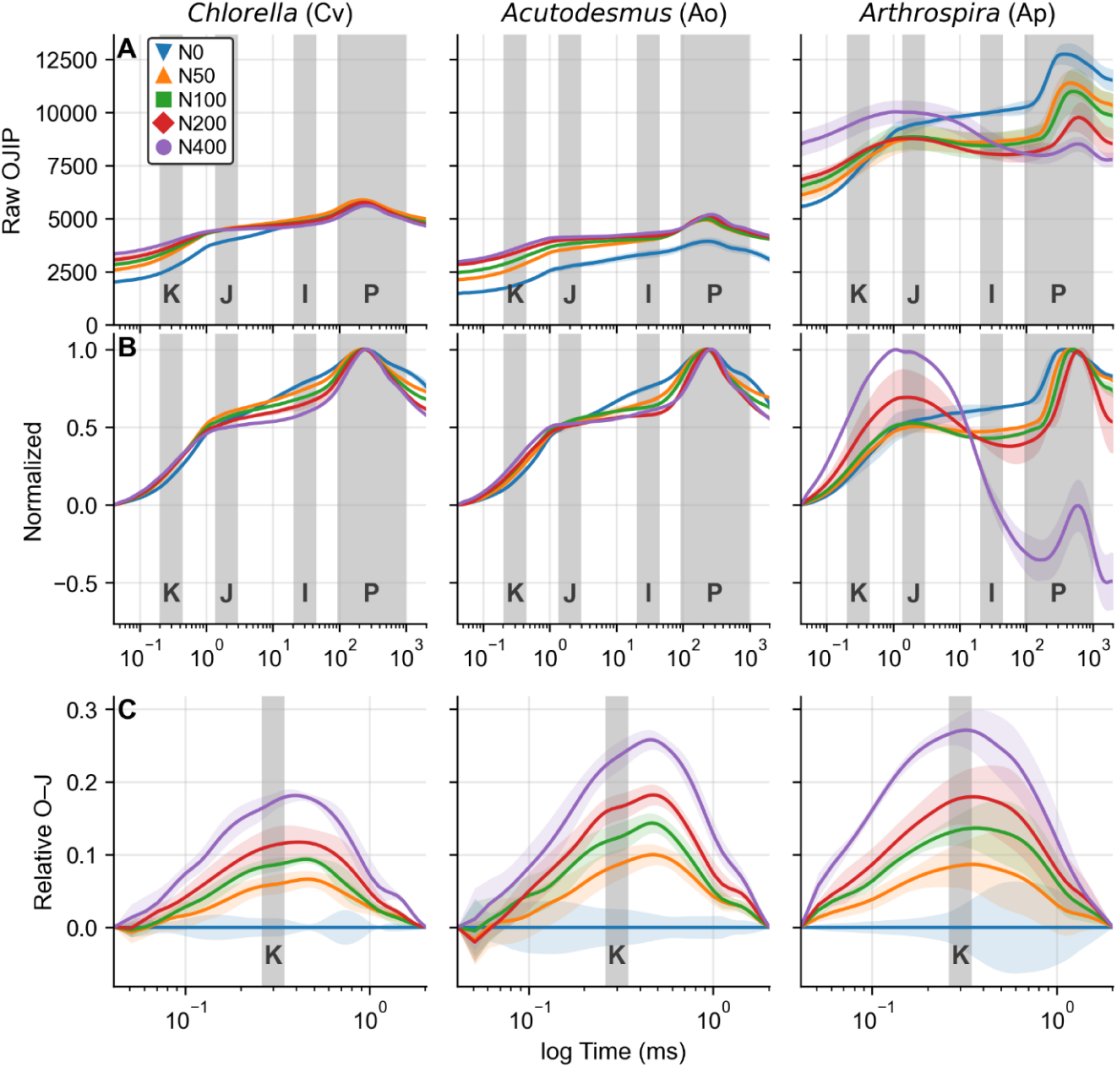
OJIP curves of the three species at Time 0. (A) Raw OJIP curves; note that *Arthrospira* was measured with a different wavelength, inducing higher intensity. (B) Double-normalized OJIP curves at *F*_m_ and *F*_0_. (C) Double-normalized curve between O and J, showing the K-band at the middle. Shaded colored envelopes show the standard deviation of triplicates.

Despite the clear effect of NH_3_ on *F*_0_ increase, such a change in raw fluorescence values cannot be used for the detection of NH_3_ toxicity directly, as absolute fluorescence intensities highly vary with biomass density and excitation light intensity. Therefore, normalized values should be employed to explore the useful parameters. While *F*_0_/*F*_m_ is a convenient normalized index to capture the *F*_0_ increase, it is also strongly influenced by the reduction of *F*_m_. To utilize the rise of *F*_0_ as an indicator, more precise normalization, such as on a chlorophyll content basis, would be required, but this is difficult to achieve during field sampling. Thus, alternative normalized parameters must be sought out.

Normalized parameters can be sought out in the OJIP curve double-normalized at *F*_m_ (P peak) and *F*_0_ (O). Here, an increase in the K step and a reduction in the I step were observed in NH_3_-inhibited cultures (Figure 3B). The increase of the K step, which is more easily visible in the relative O–J curves (Figure 3C), has been frequently associated with donor-side perturbation, including impairment of electron donation from the OEC.^37,61^ The steeper early O–J rise, reflected by parameters such as *M*_0_ and *V*_k_/*V*_j_, is interpreted here as an accelerated closure of PSII centers and an altered donor/acceptor-side balance. In parallel, the reduced I step suggests that the balance between PQ-pool reduction by PSII and its reoxidation shifted toward the latter, thereby limiting the accumulation of reduced PQ states during the J–I phase.^12^

Taken together, these three responses—higher *F*_0_, enhanced K step, and suppressed J–I rise—indicate that NH_3_ primarily impaired PSII function, likely through a combination of donor-side perturbation and altered QA/PQ redox dynamics. This interpretation is consistent with previous studies showing that ammonia can promote PSII photodamage,^60^ including effects linked to the OEC,^59^ and can also disturb ΔpH-dependent coupling processes.^22^

However, a single parameter such as *V*_k_/*V*_j_ or *F*_v_/*F*_m_ is not stable enough to be used for predicting NH_3_ toxicity, as these parameters can also shift with other factors, such as light intensity (e.g., Figure 1B and Figure S4). Instead, a combination of several parameters to extract the NH_3_-specific changes through multivariable analyses can be an alternative method. Since the OJIP curve can represent changes in different parts of the photosynthetic pathway, NH_3_-specific information may be extracted from various parameters.

### 3.3 Multivariable Modeling of NH_3_ Toxicity

The aim of this section is to establish a minimal yet transferable method to estimate free ammonia (NH_3_) concentrations from chlorophyll fluorescence transients (the OJIP curve). Three multivariable analyses were compared and evaluated to elucidate whether multivariable models based on the OJIP fluorescence rise can provide robust predictions across related algal taxa and under different culture conditions. In addition to accuracy, we attempted to clarify operational boundaries, with which the created models can be objectively judged as to whether they can be applied to a new observation.

#### 3.3.1 Comparison of Multivariable and Sparse Regression Models

Canonical Analysis of Principal Coordinates (CAP), when implemented as canonical discriminant analysis (CDA), can categorize samples by types of toxicity, as has been demonstrated in ecological applications.^18,42^ The categorization by CAP was superior in interspecies categorization by Ridge and Lasso (Table 2), most likely because it utilizes multidimensional space for categorization.^49^

**Table 2:** Overall Classification Accuracy of NH_3_ Levels through Discrimination Analysis with Models Trained on *Chlorella* Samples

Models	*Chlorella* ^a^	*Acutodesmus*	*Arthrospira*
CAP (CDA)^b^	81.0%	82.5%	28.6%
Ridge	93.7%	58.7%	0%
Lasso	87.3%	69.8%	5.3%

a Because the model was trained and validated on the same *Chlorella* data set, the reported accuracy represents an internal reference rather than an independent validation.

b CDA: canonical discrimination analysis.

However, categorization is not the best approach to assess the intensity of NH_3_ toxicity as it cannot distinguish levels of toxicity. In regard to the linear regression approach, canonical correlation analysis (CCorA) seemed to have lost its advantage of multidimensional space, and superior results were observed with Lasso analysis in interspecies prediction (Table 3). Both CCorA and Ridge regression provided high apparent accuracy within species but performed poorly when applied to unseen species, probably because of the retention of all variables, which hinders extrapolation across correlated feature blocks. Similarly, tree-based ensemble regressors (random forest and gradient boosting) achieved high apparent accuracy within species but showed limited transferability to unseen species, reflecting their weaker extrapolation capacity. In particular, while linear regressions (Ridge and Lasso) maintained strong correlations (*r*) for *Arthrospira*, the tree-based models showed markedly lower predictive strength, further indicating that linear sparse methods generalize better to new data. By contrast, Lasso regression yielded an accuracy comparable to Ridge regression within species while achieving superior generalization across species. Lasso accomplished this by reducing coefficients of less informative variables to zero, thereby emphasizing a smaller subset of physiologically interpretable predictors. As a form of sparse regression, Lasso preferentially retains core fluorescence features while discarding redundant ones, thereby enhancing cross-species generalization. On the basis of these comparisons, Lasso regression was selected as the primary modeling approach in this study.

**Table 3:** Metrics of Regression Analyses Using Models Trained on *Chlorella*

	*Chlorella* ^a^		*Acutodesmus*		*Arthrospira*	
Models	*R* ^2^	*r*	*R* ^2^	*r*	*R* ^2^	*r*
CAP (CCorA)^b^	0.78	0.88	0.62	0.87	0.014	0.20
Ridge	0.94	0.97	0.86	0.93	–91.6	0.96
Lasso	0.93	0.97	0.87	0.95	–107	0.95
Random forest	0.99	1.00	0.80	0.97	–7.43	0.48
Gradient boosting	1.00	1.00	0.80	0.92	–7.47	0.74

a Because the model was trained and validated on the same *Chlorella* data set, the reported accuracy represents an internal reference rather than an independent validation.

b CCorA: canonical correlation analysis.

#### 3.3.2 Lasso Prediction Models: Model Training and Interspecies Versatility

Lasso regression models were trained with cross-validation to identify stable predictive features of the OJIP transient and evaluate transferability across species. Predictions were assessed both within species and across species, and results are presented as out-of-fold validation on the shaded diagonal panels of Figure 4A and as cross-species applications on the off-diagonal panels.

**Figure 4:**
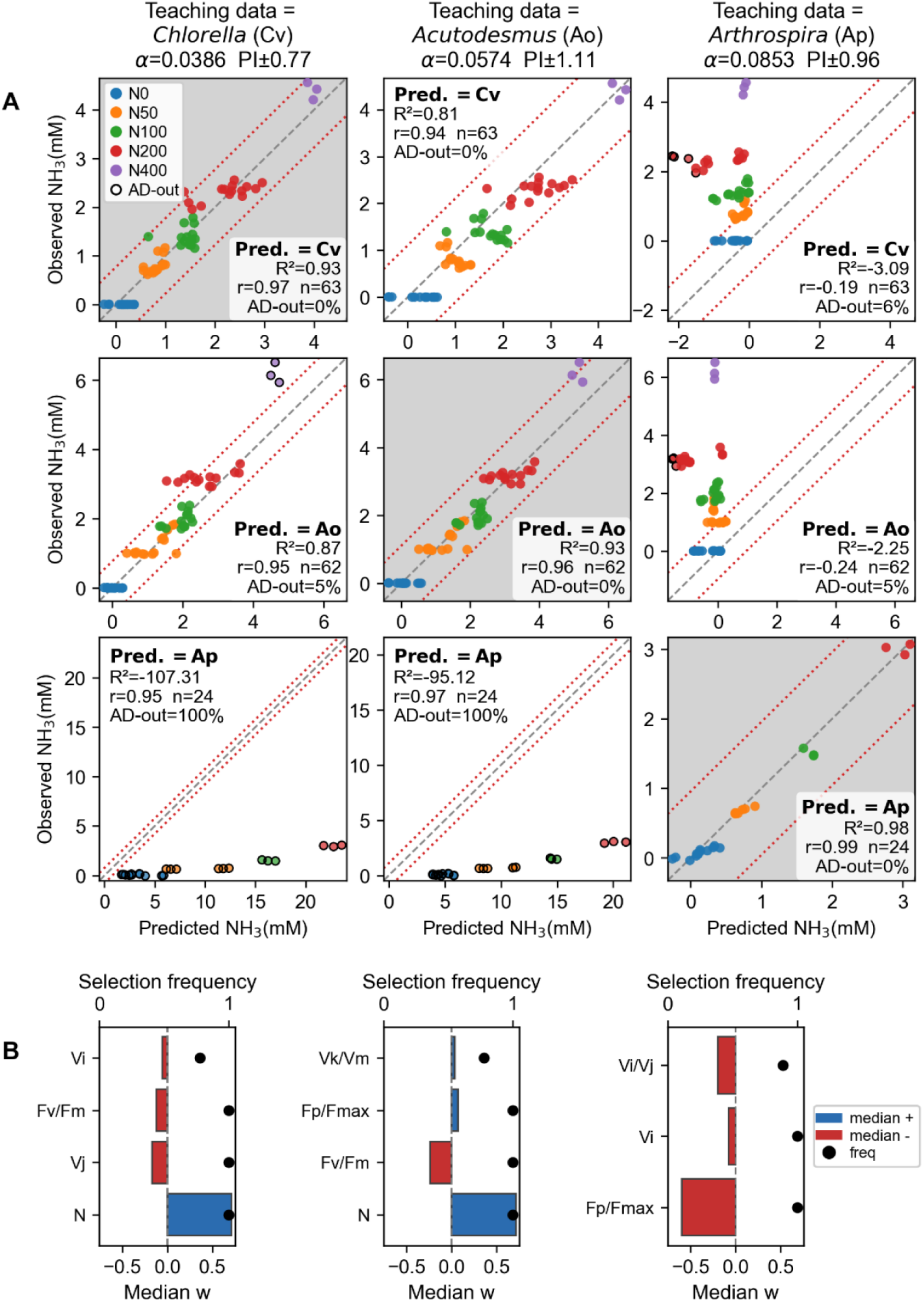
Model training and interspecies validation of Lasso regression. (A) Cross-species nested cross-validation scatter matrix (teach × predict). Shaded diagonal blocks are self-species (e.g., Cv → Cv). Dashed lines are 1:1 line; dotted red lines are 97.5% prediction interval. Applicability-domain (AD) out-of-domain samples are shown in a solid black outline. (B) Variable selection frequency with median absolute coefficient (|*w*|) (blue circles).

Within-species validation demonstrated high prediction accuracy for all three taxa, with relatively narrow prediction intervals and stable feature selection across folds (Figure 4A, diagonal; Figure 4B), yielding *R* ^2^ = 0.93–0.98 and prediction intervals (PIs) of ±0.77–1.11 mM. This performance is notable given that *F*_v_/*F*_m_ alone was influenced by early photoinhibition during culture acclimation, as indicated by the decrease observed even in the control cultures (Figure 1B; see Supporting Information Section 4). In other words, the model did not rely solely on the absolute level of *F*_v_/*F*_m_, but on the broader OJIP fingerprint. These results suggest that the multivariable approach was less sensitive to transient photoinhibition than single-parameter monitoring and therefore provided more robust NH_3_ prediction under the tested conditions.

Several core features (fluorescent parameters), notably the turnover number of QA reduction (*N*), were consistently selected across folds and species. Together with *S*_m_, *N* belongs to a group of descriptors that summarize the multiturnover capacity of the electron-acceptor side beyond QA before *F*_m_ is reached.^62^ These variables do not identify a single primary site of NH_3_ action; rather, they provide integrated descriptors of electron-acceptor-side behavior, including the balance of electron flow through PSII and downstream acceptors such as the PQ pool.^62,63^ Their repeated selection suggests that NH_3_ prediction depended not only on proximal PSII-related perturbation but also on broader changes in acceptor-side behavior. In this context, the combination of lower *V*_i_ and higher *N* is consistent with a shift of the acceptor side toward a more oxidized state, possibly reflecting altered photosynthetic control and energetic coupling through reduced ΔpH,^64^ though this interpretation remains indirect and does not establish a single validated mechanism.

Cross-species transfer between the two green algae, *Chlorella* and *Acutodesmus*, was successful, but the transfer between *Chlorella* and *Acutodesmus* was asymmetric. The models trained on *Chlorella* generalized better to *Acutodesmus* (*R* ^2^ = 0.87) than the reverse (*R* ^2^ = 0.81). This asymmetry likely arises from broader variability and better feature space coverage in the *Chlorella* training data, which included both photoinhibition and recovery trajectories. This variability allowed the Lasso model to identify stable OJIP parameters, which enhanced the NH_3_ prediction under the influence of photoinhibition. Consistent with this interpretation, applying the *Acutodesmus*-trained model to *Chlorella* produced greater dispersion (Figure 4A, upper middle). Accordingly, the *Chlorella* *-*trained model is more inclusive and better suited to fluctuating samples because of the diversity in the training sample.

Models trained on *Arthrospira* did not transfer to green algae (Figure 4A, right), consistent with the selection of variables that were specific to the fluorescence characteristics of this cyanobacterium, including the custom ratio *F*_p_/*F*_max_. This parameter is interpreted here as a species-specific descriptor rather than a universal cyanobacterial marker. Its repeated selection in *Arthrospira* may reflect the fact that cyanobacterial fluorescence kinetics are shaped not only by PSII photochemistry but also by phycobilisome-associated excitation transfer, state transitions, and interactions between photosynthetic and respiratory electron transport.^27^ These processes can alter the relative prominence of the J region and the terminal peak in ways that are not directly comparable to those of the green algal curves. Conversely, models trained on green algae preserved a high correlation (*r*) between predicted and observed NH_3_ concentrations in *Arthrospira* (Figure 4A, left bottom), but the absolute errors remained large. This pattern suggests that some broad NH_3_-related stress signatures may be shared across phototrophic taxa, whereas the quantitative conversion of OJIP features into absolute NH_3_ concentrations is not directly transferable from green algae to cyanobacteria. Because cyanobacterial fluorescence kinetics are influenced by phycobilisome excitation transfer, state transitions, and coupling between photosynthetic and respiratory electron transport, cyanobacterial deployment should use separate calibration; species- or lineage-specific calibration may be required for taxa beyond *Arthrospira*. Overall, the results indicate that sparse regression enhances generalization by suppressing overfitting to the correlated blocks of variables.

#### 3.3.3 External Validation and Uncertainty Quantification

To evaluate robustness under distribution shift, models on the NH_3_ toxicity tolerance test (Culture 1) were trained and applied to the independent validation culture (Culture 2), which differed in growth trajectories and NH_3_ dynamics. The models were evaluated based on whether each sample falls within the applicability domain (AD) and prediction interval (PI), as well as standard metrics (*R* ^2^ and RMSE).

Prediction performance remained high for *Chlorella* and *Arthrospira*, with *R* ^2^ values of 0.87 and RMSE of 0.55–0.84 mM (Figure 5A,C). These results demonstrate that the proposed sparse regression approach retains predictive validity under varying exposure conditions, a prerequisite for reliable application in hazard monitoring. The validation of *Chlorella* had low AD-out and PI-out ratios of 0% and 23%, despite the difference in the culture condition, indicating that the model was inclusive. On the other hand, that of *Arthrospira* showed low AD-out (0%) but high PI-out (38%), indicating a highly inclusive but less accurate model. This lower accuracy was induced by the small number of samples in the teaching data after quality assurance (*n* = 30). Notably, the model successfully captured time-dependent changes in NH_3_ concentration, demonstrating that chlorophyll fluorescence can dynamically track NH_3_ variation during algal cultivation. For example, in *Chlorella* at low nitrogen concentrations (N50), the actual observed NH_3_ concentrations rose from approximately 0.7 mM at inoculation to 1.8 mM after 14 h owing to the pH-driven shift in NH_3_/NH_4_^+^ equilibrium, and the predicted values paralleled this shift of NH_3_ (Figure 5A). Similarly, in *Arthrospira*, decreases in total ammoniacal nitrogen during the same interval were mirrored by declines in predicted NH_3_, approaching zero after 14 h (Figure 5C). These changes in the observed NH_3_ concentrations were well predicted by the model, further ensuring the ability of chlorophyll fluorescence to monitor the change of the culture. This capacity supports its potential for real-time monitoring in controlled phototrophic cultivation and treatment systems. Validation in real wastewater containing additional chemical and biological interferences remains future work.

**Figure 5:**
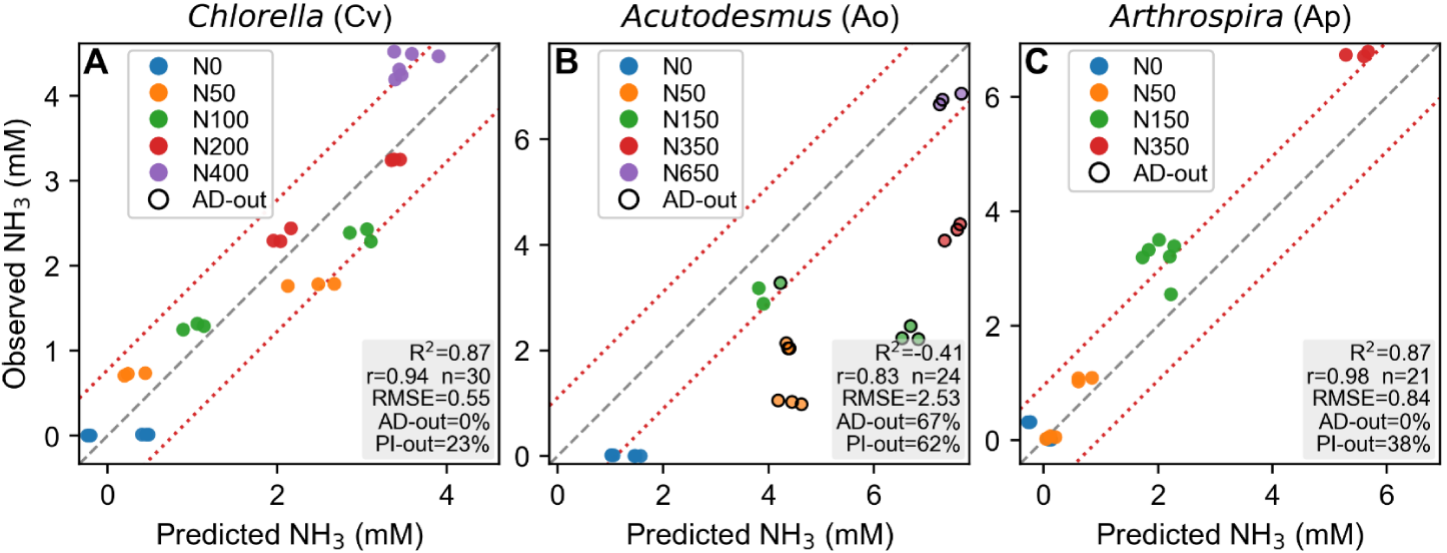
External validation on Culture 2. Predicted vs observed NH_3_ for (A) *Chlorella* (Cv), (B) *Acutodesmus* (Ao), and (C) *Arthrospira* (Ap); the dashed black line is 1:1 line, and the dotted red lines are 97.5% prediction interval. Applicability-domain (AD) out-of-domain samples are shown in a solid black outline. AD and PI limits were calibrated on Culture 1 training data and applied unchanged to Culture 2; no validation-set recalibration was performed.

By contrast, predictions for *Acutodesmus* showed larger errors and a higher fraction of samples outside the AD (Figure 5B). This pattern reflects both the relative homogeneity of *Acutodesmus* training data, which limited model flexibility, and the deviation of the fluorescence data in Culture 2, partially owing to the high NH_3_ levels. Nevertheless, the explicit reporting of AD and PI enabled a clear diagnosis of failure modes, distinguishing cases in which fluorescence signals fell outside the training distribution from cases in which uncertainty alone was large. These results demonstrate that chlorophyll fluorescence–based prediction of NH_3_ can remain reliable under substantial variation in culture conditions, as shown by two campaigns differing by more than 10-fold in cell density and exhibiting distinct NH_3_ ranges. While many *Acutodesmus* samples were AD-out, the AD/PI framework effectively prevented false confidence and retraining with the AD-out data improved generality. Because changes in cell density inherently modify the light environment within the culture, these findings indicate that the model is robust against moderate shifts in the light regime, cell density, and NH_3_ concentration, although broader training diversity will be required for robust deployment under more variable wastewater conditions.

There are several ways to improve the models, such as by incorporating other fluorescent protocols and increasing the number and variety of samples. In regard to other fluorescent protocols, incorporation of the data from the light curve protocol (LC2) was tested but resulted in only minor improvements in some cases (cf. Supporting Information Section 9). This result confirms that OJIP contains a variety of information on the photosystems, which is enough to predict NH_3_ toxicity from just a couple of seconds of measurement. By increasing the number and variety of teaching samples, models created by the OJIP can further become versatile.

To confirm whether the expansion of sample variety can lead to a more versatile model, an inclusive model that pooled all green algal data from both Culture 1 and Culture 2 was constructed. Internal validation of this combined data set demonstrated expanded applicability-domain coverage while maintaining reasonable prediction-interval widths (Figure 6A). When applied illustratively to *Acutodesmus* from Culture 2, the combined model retained all samples within both AD and PI boundaries (Figure 6B). Although this approach does not replace comprehensive external validation, it demonstrates that combining diverse training data with strict quality control can yield models suitable for the real-time monitoring of algal cultures and wastewater treatment systems.

**Figure 6:**
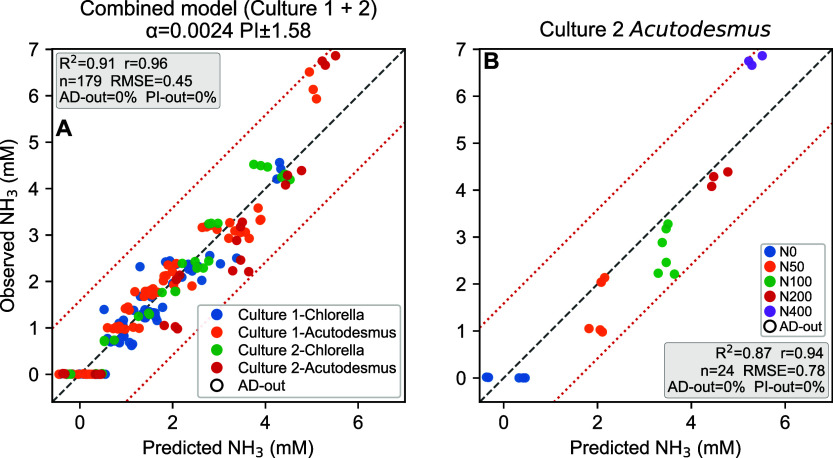
Demonstration of an inclusive model with combined training by *Chlorella* and *Acutodesmus* data from both Culture 1 and Culture 2. (A) Final Lasso model (α chosen via grouped nested cross-validation) predictions on all combined data (Pred vs Obs) colored by Culture-Species (Culture 1/2 × *Chlorella*/*Acutodesmus*); the black line is 1:1 line; the dashed red lines are ± 95% prediction interval (PI); applicability-domain (AD) out-of-domain samples are shown in a solid black outline. (B) The same combined model applied to Culture 2 *Acutodesmus*, showing PI coverage and AD-out fraction. For this pooled-data demonstration, AD and PI limits were calibrated from the combined green-algal training set and then held fixed for the displayed predictions.

#### 3.3.4 Deployment Workflow: From Model Development to Operational Use

For practical deployment in algal systems and environmental hazard monitoring, a concise workflow linking data quality, standardized preprocessing, and explicit operational boundaries is proposed (Figure 7). During model development, fluorescence data should be collected under a fixed measurement protocol to minimize the variability. Quality control removes samples with noise, truncation, or saturation, and features were scaled by using parameters from the training set. The Lasso regression model is then optimized by nested cross-validation, and residuals are used to calibrate the prediction intervals. The applicability domain is defined in the fluorescence feature space, yielding a fixed package of scaler, model, AD definition, and PI calibration.

**Figure 7:**
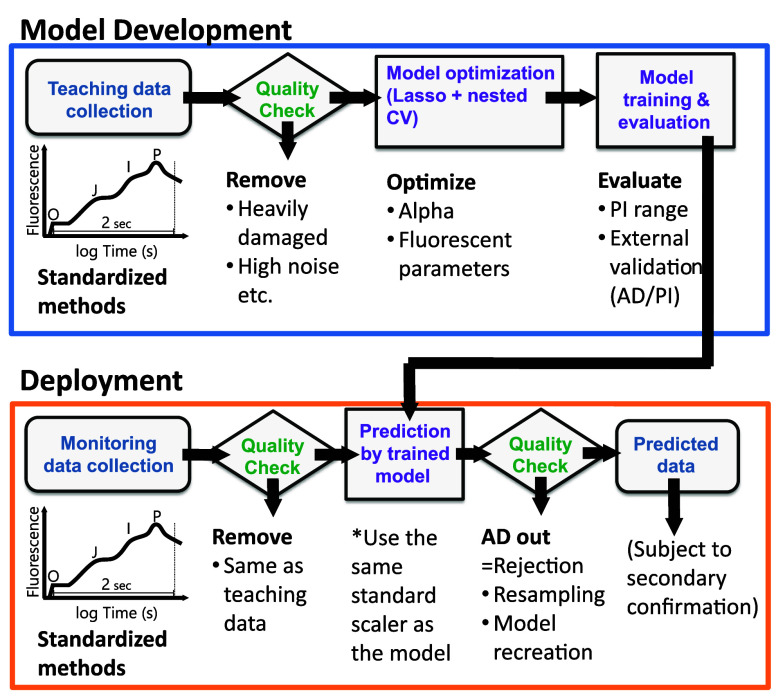
Suggested model development and deployment workflow for NH_3_ prediction using chlorophyll fluorescence. CV: cross-validation; AD: applicability domain; PI: prediction interval.

In deployment, this package is applied to new monitoring data by using the same steps. Measurements must follow identical standards since differences in excitation wavelength, saturation pulse, or detector settings can shift samples outside the applicability domain. Raw traces undergo the same quality checks, features are standardized, and each sample is screened against AD. Out-of-domain inputs are rejected, while in-domain predictions are provided with their PIs. Wide intervals flag high uncertainty and prompt confirmation before action.

As shown in Figure 7, this workflow clarifies failure modes—measurement errors, domain extrapolation, or statistical uncertainty—and ensures transparent operation. Logging of AD and PI decisions supports drift monitoring and guides retraining when the operating space expands. When drift is detected, NH_3_ concentrations for representative AD-out samples are assayed and the resulting labeled data are appended to the training set, followed by controlled model retraining. This loop can be automated via periodic reference measurements and scheduled updates, closing the cycle between deployment and training. By enforcing standardized protocols and explicit boundaries, fluorescence-based NH_3_ prediction can serve as a reagent-free biosensing method for real-time hazard monitoring in algal and wastewater environments. This framework is consistent with best practices in predictive environmental modeling.^65–68^ By following these principles, chlorophyll fluorescence–based NH_3_ prediction can be applied safely and reproducibly to real-time monitoring of algal cultivation and wastewater treatment systems.

### 3.4 Implications, Limitations, and Outlook for Environmental Hazard Assessment

From the perspective of environmental toxicology, this study provides a mechanistically interpretable and transferable approach to quantifying ammonia-induced stress through optical biosensing. The present study shows that OJIP fluorescence transients, when analyzed with sparse regression and explicit boundary diagnostics, can provide a practical method for estimating free-ammonia concentrations in phototrophic systems. This approach requires only seconds of nondestructive measurement, uses open-source computational tools, and avoids the need for chemical reagents or expensive proprietary equipment. Although single fluorescence indices such as *F*_v_/*F*_m_ or *V*_k_/*V*_j_ are not uniquely specific to NH_3_,^39^ the multivariate OJIP approach combines complementary information from different parts of the fluorescence transients. Early-phase descriptors such as *M*_0_ and *V*_k_/*V*_j_ captured proximal changes related to PSII donor-side perturbation, whereas later and integrated descriptors such as *S*_m_ and *N* reflected broader changes in acceptor-side redox behavior and multiturnover electron transport.^12,62^ These latter variables are not interpreted here as direct reporters of proton gradient disruption. Rather, they are discussed as integrated descriptors of downstream electron-acceptor behavior and energetic coupling, which may be influenced when NH_3_ perturbs photosynthesis at multiple levels. Under the controlled conditions tested here, this broader fluorescence fingerprint enabled NH_3_ prediction to be more effective than any single parameter alone. In a stress response that likely affects multiple parts of the photosynthetic system, the predictive importance of these integrated descriptors is, therefore, biologically plausible. The resulting models were most promising as a basis for bioprocess monitoring that receives a low-millimolar NH_3_ range, particularly in algal cultivation or treatment systems where the dominant phototroph is known and the measurement protocol can be standardized. In such settings, applicability-domain and prediction-interval diagnostics allow operators to automate decisions such as whether data can be trusted, require confirmation, or should be discarded. By contrast, direct application as a broad bioindication tool in natural or mixed communities would require substantially wider validation because multiple concurrent stressors and taxonomic variability may alter the fluorescence response independently of NH_3_.

A second possible use case is biotesting with a standardized sentinel strain, which can be applied to estimate NH_3_ in the environment. This approach may be advantageous compared with bioindication because it reduces taxonomic variability and simplifies calibration, but it would require controlled exposure conditions and explicit evaluation of confounding stressors. The present study was not designed to establish a single sentinel-species protocol; rather, it was intended to determine whether NH_3_-responsive fluorescence signatures could be quantified across multiple phototrophic systems and whether cross-taxon calibration was feasible.

Nonetheless, the present study has several limitations that should be acknowledged. A key limitation is that the present models were trained under controlled NH_3_ exposure and were not challenged with other chemical or physical stresses that may induce overlapping fluorescence responses. The current framework should therefore be regarded as a controlled-condition NH_3_ prediction method rather than a universally stress-specific classifier. Future work should test specificity explicitly through multifactor experiments involving light, temperature, salinity, metals, herbicides, and mixed matrices such as real wastewater. This limitation is particularly important for cyanobacteria, whose fluorescence kinetics are influenced by phycobilisome-associated excitation transfer, state transitions, and the coupling between photosynthetic and respiratory electron transport.^26^ Accordingly, the present results should not be interpreted as supporting the direct transfer of green-algal OJIP coefficients or calibration functions to cyanobacteria. For operational use, cyanobacterial systems should first be calibrated and validated separately from those of green algae. In the present study, the *Arthrospira* data show that the fluorescence-to-NH_3_ framework can be calibrated within one cyanobacterial system under controlled conditions but does not establish a universal cyanobacterial model.

Future work should therefore distinguish protocol standardization from model transferability. A broadly similar measurement and preprocessing protocol, for example, one based on a common red measuring light source where instrumentally feasible, may provide a useful benchmarking framework across green algae and cyanobacteria. However, because cyanobacterial fluorescence is strongly shaped by lineage- and species-dependent state transitions and phycobilisome dynamics, absolute NH_3_ prediction will likely require species- or lineage-specific calibration, until cross-cyanobacterial validation demonstrates otherwise.

In addition, external validation was limited to two culture campaigns at one facility, and broader testing across instruments, light protocols, and sites is required. Differences in the excitation wavelength, saturation pulse intensity, and instrument sensitivity may further affect model transferability and must be standardized before operational deployment.

An additional limitation is that mechanistic interpretation remains indirect. The present study did not include complementary assays such as proton-flux measurements, ATPase activity, OEC activity, or Mn content analysis. Accordingly, the selected fluorescence features should not be interpreted as definitive markers of the specific inhibition sites. Instead, they indicate physiologically plausible regions of perturbation that require direct mechanistic validation in future studies. Future work should prioritize method standardization and validation across multiple instruments, multiple culture conditions, and sites to expand the training domain. A priority next step is validation in real wastewater and digestate-derived matrices, where additional contaminants and variable background chemistry may interfere with the fluorescence-based NH_3_ prediction. In the current study, the method was shown to distinguish NH_3_ toxicity from light-induced reductions in *F*_v_/*F*_m_ during the early acclimation period of the cultures, highlighting the advantage of multivariable OJIP analysis over single-stress-sensitive indices. However, potential interference from other stressors (e.g., wastewater matrices, heat, salinity, and metals) should be confirmed through controlled multifactor experiments to test specificity. Combining fluorescence monitoring with more direct periodic measurements of NH_3_ and targeted mechanistic assays would further reduce the risk of misinterpretation.

With these developments, fluorescence-based NH_3_ monitoring has the potential to evolve into a biosensor platform for algal systems and potentially in broad aquatic systems. For example, pairing with highly sensitive taxa such as the marine green alga *Nephroselmis pyriformis*, which has an EC_50_ for NH_3_ in the micromolar range,^69^ may enable highly sensitive biosensors. Upon such an improvement in model training and validation, chlorophyll fluorescence combined with multivariable analyses may eventually enable the detection and quantification of ammonia in algal cultures, wastewater treatment facilities, industrial effluents, and natural waters.

This study demonstrated that rapid chlorophyll fluorescence transients analyzed with sparse regression can provide robust predictions of free-ammonia concentrations in algal systems. Among the tested models, Lasso regression yielded the most transferable performance, accurately predicting NH_3_ within each tested taxon and across the two green algal taxa, while cyanobacterial applications require separate and potentially species- or lineage-specific calibrations. The integration of AD and PI diagnostics ensures transparent uncertainty quantification, enabling safe and reproducible deployment in real-time hazard monitoring. This framework highlights the potential of interpretable machine learning to establish reagent-free biosensing tools for environmental risk assessment.

## Supplementary Material



## Data Availability

Curated data are available on the open archive Zenodo (10.5281/zenodo.17358670). The Python code along with example data is also available on GitHub (https://github.com/MasaK2010/OJIPLasso) and on Zenodo (10.5281/zenodo.17398400), which is subject to minor updates.

## Notes

Additional methods for fluorescence preprocessing, quality control, grouped nested cross-validation, standardization, and applicability-domain/prediction-interval calculations; comprehensive OJIP-derived parameter definitions and ablation tests; representative OJIP curves; temporal parameter changes; *F*_v_/*F*_m_ correlation analyses; α optimization; validation-culture data; LC2 comparison; and pH time-course data (PDF)
