# Supplementary material for: Reagent-Free Prediction of Free-Ammonia Toxicity in Algal Systems Using Chlorophyll Fluorescence Transients and Interpretable Sparse Regression
Source: Environ Sci Technol. 2026 Jun 15;60(25):17600–14. doi: 10.1021/acs.est.5c18003 (PMC13325865; doi:10.1021/acs.est.5c18003)
Supplement: Supplementary file 1 [file es5c18003_si_001.pdf]

**Supporting Information**

# Reagent-free prediction of free-ammonia toxicity in algal systems using chlorophyll fluorescence transients and interpretable sparse regression


Masatoshi Kishi [a,b,c*], Panagiota Karachaliou [d], Tetsuichi Fujiki [c], Maki Noguchi Aita [c], Raúl Muñoz [a,b*]

[a] Institute of Sustainable Processes, University of Valladolid, 47011, Valladolid, Spain.
[b] Department of Chemical Engineering and Environmental Technology, School of Industrial Engineering, University of Valladolid, Dr. Mergelina s/n., Valladolid 47011, Spain.
[c] Research Institute for Global Change, Japan Agency for Marine-Earth Science and Technology, 2-15 Natsushima-cho, Yokosuka, Kanagawa, 237-0061, Japan.
[d] Department of Chemical Engineering, Cyprus University of Technology, 57 Anexartisias Str., 3603 Limassol, Cyprus
*Corresponding author: kishi.masatoshi@uva.es (M. Kishi); raul.munoz.torre@uva.es (R. Muñoz)


**Contents summary:** 15 pages, 8 figures, 2 tables.

## 1. Data processing

### a. Overview

Fluorescence data were processed in the following sequence: logarithmic resampling of the OJIP transients, spline smoothing, normalization, parameter calculation, and finally quality control (QC). This pipeline ensured that kinetic features were preserved, parameters were extracted consistently, and unreliable data were excluded.

### b. List of parameters and their ablation through prediction trials

Table S1 provides a comprehensive list of all OJIP-derived parameters considered in this study. The table includes raw fluorescence points (Fo, Fj, Fi, Fm), relative variable fluorescence values (Vk, Vj, Vi), derived kinetic indices ($M_o$, Sm, N), performance indices (PIabs), and fluxes per reaction center (ABS/RC, $TR_o$/RC, $ET_o$/RC, $DI_o$/RC). For each parameter, the mathematical definition, and photosynthetic significance are given, along with primary references (Kalaji et al., 2016; Stirbet & Govindjee, 2011; Strasser et al., 2000).

- **Relative variable fluorescence:** $V_k$, $V_j$, $V_i$
- **Kinetic indices:** $M_o$, $S_m$, N
- **Quantum yields and efficiencies:** $\varphi P_o$, $\varphi E_o$, $\psi E_o$
- **Energy fluxes per RC:** ABS/RC, $TR_o$/RC, $ET_o$/RC, $DI_o$/RC
- **Performance indices:** $PI_{abs}$,

**Table S1**. List of parameters obtained in this study and their physiological significance.

| Parameter | Definition | Formula | Physiological significance |
|---|---|---|---|
| **$F_0$** | Minimal fluorescence | $F_o$ | All PSII RCs open |
| **$F_m$** | Maximal fluorescence | $F_m$ | All PSII RCs closed |
| **$F_m/F_0$** | Fluorescence ratio between minimal ($F_o$) and maximal fluorescence (Fm) | $\frac{F_m}{F_o}$ | PSII antenna efficiency |

| Parameter | Definition | Formula | Physiological significance |
|---|---|---|---|
| **$F_v/F_0$** | Variable fluorescence normalized to $F_0$ | $\frac{F_v}{F_o}=\frac{F_m-F_o}{F_o}$ | Potential activity of PSII |
| **$F_v/F_m$ ($\varphi P_0$)** | Maximum quantum yield of primary photochemistry | $\frac{F_v}{F_m}=\frac{F_m-F_o}{F_m}$ | PSII efficiency |
| **$V_k$** | Relative variable fluorescence at 300 µs | $\frac{F_{300\mu s}-F_o}{F_m-F_o}$ | OEC activity |
| **$V_j$** | Relative variable fluorescence at J step (~2–3 ms) | $\frac{F_j-F_o}{F_m-F_o}$ | QA reduction |
| **$V_i$** | Relative variable fluorescence at I step (~30 ms) | $\frac{F_i-F_o}{F_m-F_o}$ | PQ pool reduction |
| **$V_L$** | Relative variable fluorescence at L step (~150 µs)* | $\frac{F_{150\mu s}-F_o}{F_m-F_o}$ | L-band, connectivity between PSII units |
| **$V_{last}$** | Relative variable fluorescence at last time point* | $\frac{F_{t_{end}}-F_o}{F_m-F_o}$ | Saturation tendency |
| **$V_k/V_j$** | Ratio of early to J step | $\frac{V_k}{V_j}=\frac{F_{300\mu s}-F_o}{F_j-F_o}$ | OEC vs QA reduction |
| **$V_i/V_j$** | Ratio of I to J step | $\frac{V_i}{V_j}=\frac{F_i-F_o}{F_j-F_o}$ | PQ pool filling |
| **$F_0/F_m$** | Inverse of Fv/Fm | $\frac{F_o}{F_m}$ | Non-variable fluorescence |
| **$F_p/F_{max}$** | Ratio of P amplitude to max signal* | $\frac{F_p}{F_{\max}}$ | (Custom parameter to depict the elevated J peak over P peak) |
| **$M_0$** | Approximated initial slope of fluorescence rise | $M_0=\frac{4(F_{300\mu s}-F_o)}{F_m-F_o}$ | QA reduction rate |
| **Area** | Integrated area above the fluorescence curve up to $t_{Fm}$ | $\int_{t_0}^{t_{Fm}}(F_m-F(t))dt$ | Total number of electrons required to reduce the complete acceptor pool beyond QA |
| **$S_m$** | Normalized area above the fluorescence curve | $\frac{Area}{F_m-F_o}$ | Size of electron acceptor pool |
| **N** | Number of QA reduction events until Fm | $\frac{Sm\times M_0}{V_j}$ | Electron transport capacity |
| **$\varphi P_0$** | Maximum quantum yield of primary photochemistry | $\varphi_{Po}=F_v/F_m$ | PSII efficiency |

| Parameter | Definition | Formula | Physiological significance |
|---|---|---|---|
| **ψEo** | Probability that a trapped exciton moves an electron beyond $QA^{-}$ | $1 - V_j$ | QA to PQ transfer efficiency |
| **φEo** | Quantum yield of electron transport | $\phi_{Eo} = \phi_{Po} \cdot \psi_{Eo}$ | PSII electron transport |
| **φDo** | Quantum yield of energy dissipation | $\phi_{Do} = 1 - \phi_{Po}$ | Non-photochemical dissipation |
| **ABS/RC** | Absorption flux per RC | $\frac{M_0}{V_j} \cdot \frac{1}{\phi_{Po}}$ | Antenna size per RC |
| **$TR_0$/RC** | Trapped energy flux per RC | $\frac{M_0}{V_j}$ | Photochemistry at PSII |
| **$ET_0$/RC** | Electron transport flux per RC | $(TR_0/RC) \cdot (1 - V_j)$ | Electron transport beyond QA |
| **$DI_0$/RC** | Dissipated energy flux per RC | $ABS/RC - TR_0/RC$ | Heat/fluorescence dissipation |
| **$PI_{abs}$** | Performance index on absorption basis | $\frac{RC/ABS}{1 - RC/ABS} \cdot \frac{\phi_{Po}}{1 - \phi_{Po}} \cdot \frac{\psi_{Eo}}{1 - \psi_{Eo}}$ | PSII performance |
| **$P_{time}$** | Time to reach maximum fluorescence* | $t_{Fm}$ | PSII closure time |
| **$B_{av}$** | Average fraction of closed PSII reaction centers between O to P | $1 - S_m/t_{Fm}$ | Mean redox state of QA and the degree of PSII center closure |

*Custom parameter
**Abbreviations**: PSII = photosystem II; RC = reaction center; QA = primary quinone electron acceptor of PSII; PQ = plastoquinone pool (secondary electron acceptor beyond QA); OEC = oxygen evolving complex.

In order to focus on physiologically relevant OJIP features in the multivariable analysis, predictive statistics were compared among (1) All OJIP variables, (2) relative variables (e.g., Fv/Fm, Vj) + kinetics indices (e.g., Sm, N), and (3) only relative variables. As a result, while the first condition showed the highest predictive performance, the difference between (1) and (2) was minor (Table S2). On the other hand, (3) showed substantially poorer prediction performance. Thus, it was shown that kinetic indices were critical in NH3 prediction, and therefore (2) was used in this study.

**Table S2**. Ablation test of OJIP features in prediction of NH3 concentration.

| | | (1) All OJIP | (2) Relative + kinetics | (3) Only relative |
|---|---|---|---|---|
| *Chlorella* model to predict *Acutodesmus* (Table 3) | $R^2$ | 0.88 | 0.87 | 0.82 |
| | R | 0.95 | 0.95 | 0.91 |
| Combined model (Culture 1 + 2) to Culture 2 *Acutodesmus* (Fig. 6b) | $R^2$ | 0.93 | 0.87 | 0.69 |
| | RMSE (mM) | 0.57 | 0.78 | 1.19 |

List of variables selected for multivariable analyses: $V_j$, $V_i$, $F_m/F_0$, $F_v/F_0$, $F_v/F_m$, $F_p/F_{max}$, $F_0/F_m$, $M_o$, $S_m$, N, $V_L$, $V_k$, $V_k/V_j$, $V_j/V_m$, $V_k/V_m$, $V_i/V_j$.

## c. Quality control (QC) criteria

The following filters were implemented to ensure reliability of parameter datasets. Each criterion is expressed mathematically, with its biological rationale:

- **Low PSII maximum efficiency** ($F_v/F_m$)
  - Flag = True if $F_v/F_m < 0.05$ (only when $F_v/F_m$ is finite).
- **High relative noise near $V_j$**
  - Let $\sigma V_j$ be the standard deviation of the raw signal in the $V_j$ window (~2 ms, unsmoothed).
  - Flag = True if $|\sigma V_j| / (F_j - F_0)| > 0.15$ (only when the ratio is finite).
- **No obvious P-peak**

First, we defined "No normal P peak" flag as follows:
P peak candidates were searched between 0.1–1.9 s using two complementary rules:

1. Primary candidates at zero-crossings of the first derivative (D1) that are also local maxima of fluorescence;
2. Additional candidates at local maxima where D1 is near zero and the second derivative (D2) is negative (downward curvature).

If ≥1 candidate existed, we selected the one with the highest fluorescence as P.
However, this flag also contained some small P-peaks appearing from noise. To exclude such errors, we added an additional criterion for the "No obvious P-peak" filter.

3. Let $V_{RP}$ be the relative fluorescence value at the inflection point before the P rise, $V_P$ be the relarive fluorescent value at the selected P peak, and Vj be the relative fluorescence value at the J step. The trace was flagged as "No obvious P-peak" when:
   $[V_P < V_j \wedge (V_P - V_{RP}) \leq 0.2]$

- **Low $V_k$:**

Discards traces where the early O–K rise was not detectable, indicating technical error or very low cell density.

$V_k < 0.10$

# 2. OJIP curves of all data

Representative OJIP curves across all $NH_3$ treatments, after smoothing. Raw OJIP curves include all sample data without quality assurance sample filtering.

### a. Culture 1 (Fig. S1)

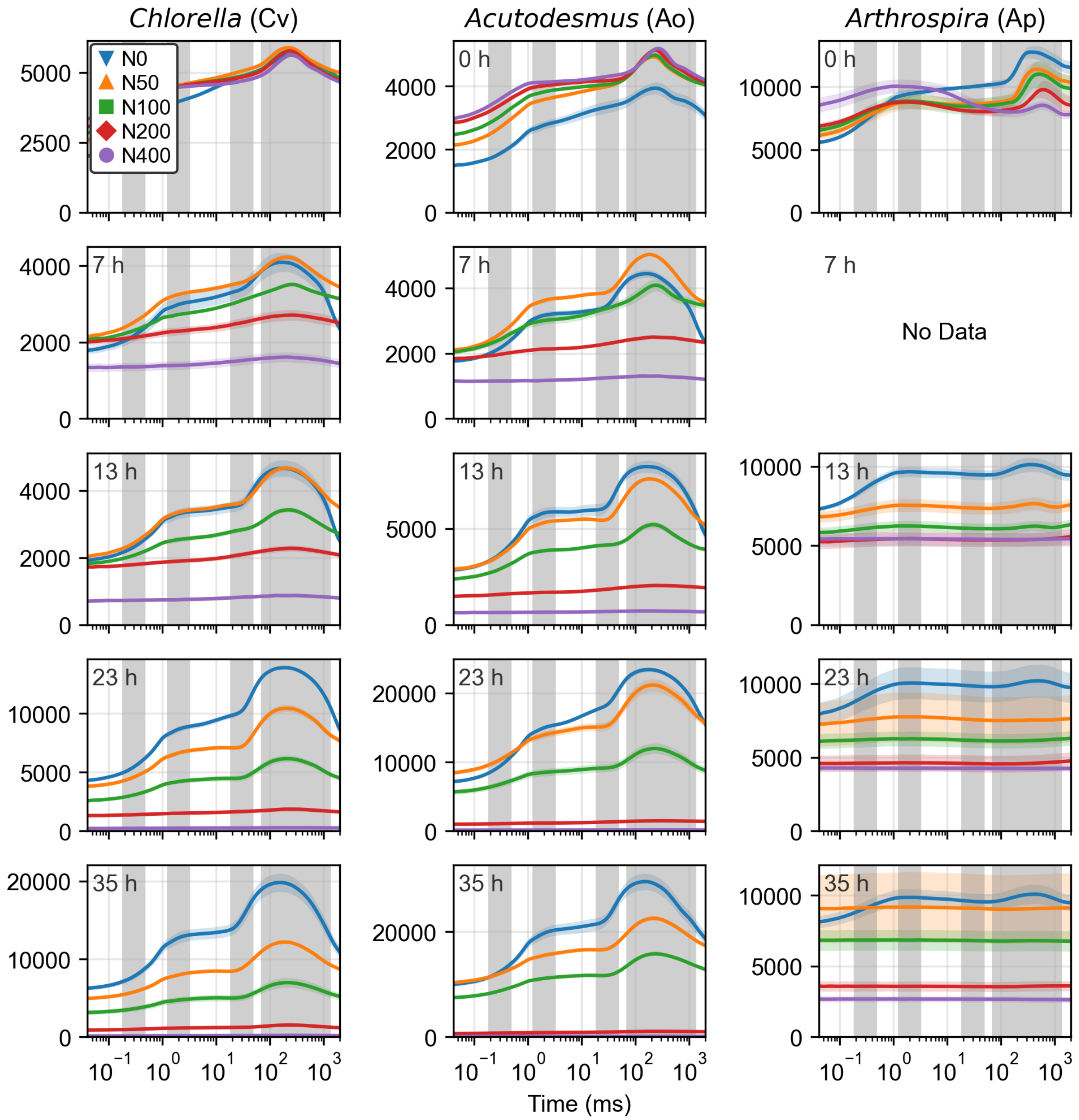


**Figure S1.** Raw baseline-corrected OJIP curves of Culture 1 by sampling time and species. Columns: *Chlorella* (Cv), *Acutodesmus* (Ao), *Arthrospira* (Ap). Rows: Sampling time (0 to 35 h). Raw baseline-corrected curves with $NH_3$ treatments colored (N0-N400). Shaded envelopes show the standard deviation of triplicates.

## b. Culture 2 (Fig. S2)

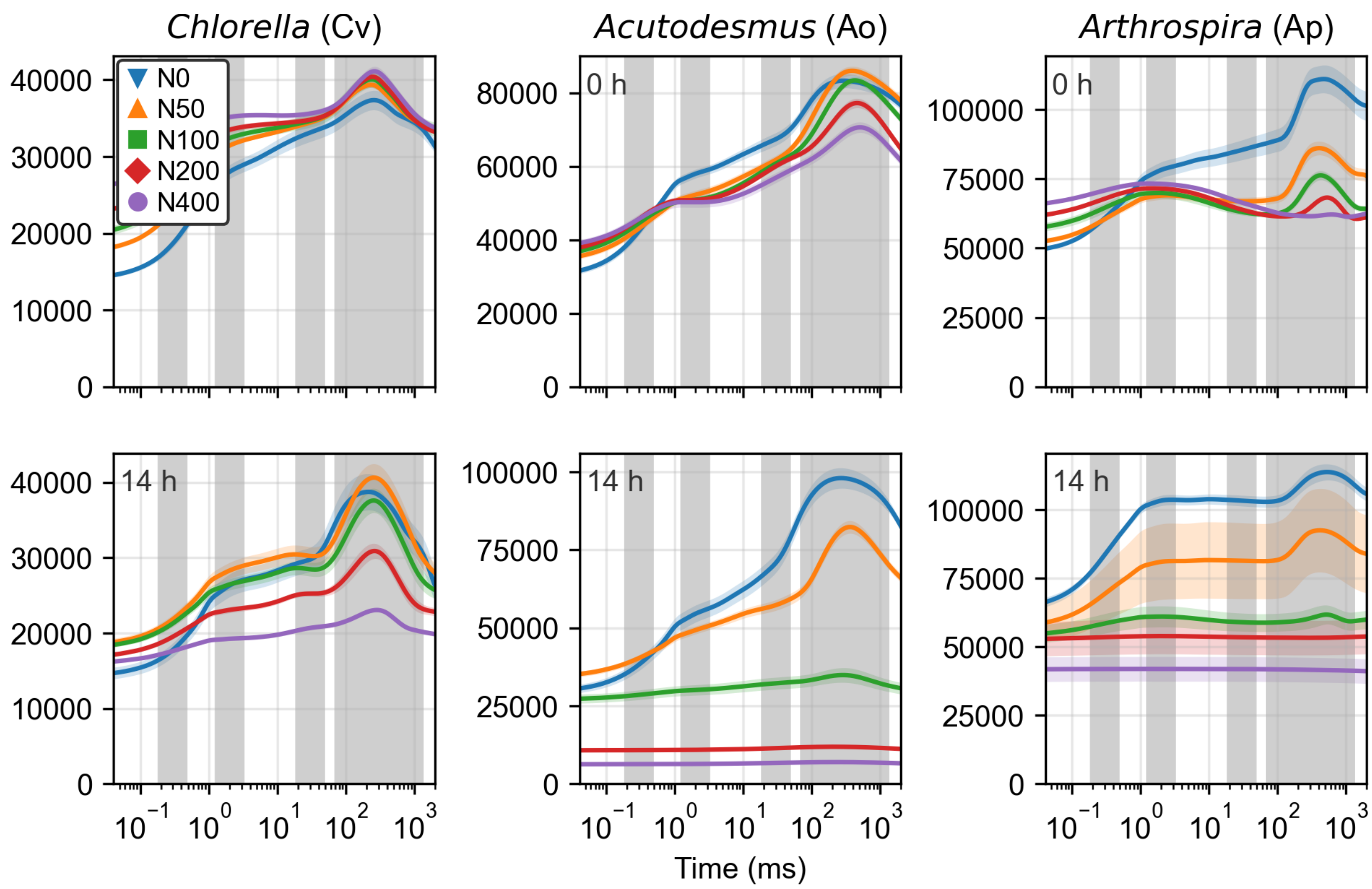


**Figure S2.** Raw baseline-corrected OJIP curves of Culture 2 by sampling time and species. Columns: *Chlorella* (Cv), *Acutodesmus* (Ao), *Arthrospira* (Ap). Rows: Sampling time (0 and 14 h). Raw baseline-corrected curves with $NH_3$ treatments colored (Cv: N0-N400; Ao/Ap: N0-N650). Shaded envelopes show the standard deviation of triplicates.

Culture 2 exhibited noticeably different kinetics compared to Culture 1, particularly in *A. obliquus* recovery experiments. These deviations resulted in more frequent classification as AD-out in model evaluation. The discrepancy highlights the sensitivity of OJIP kinetics to culture history and physiological state.

## 3. Temporal changes of parameters

Time courses (0–36 h) of selected OJIP parameters under all $NH_3$ treatments. The first two parameters showed strong correlation with $NH_3$ and temporal stability, whereas the lower three were more frequently selected in Lasso models. This indicates that model selection does not necessarily favor parameters with the highest correlation or stability, but those contributing complementary predictive information. Some parameters were shared across all species, while others were specific to *A. platensis*, likely due to its higher sensitivity to ammonia toxicity.

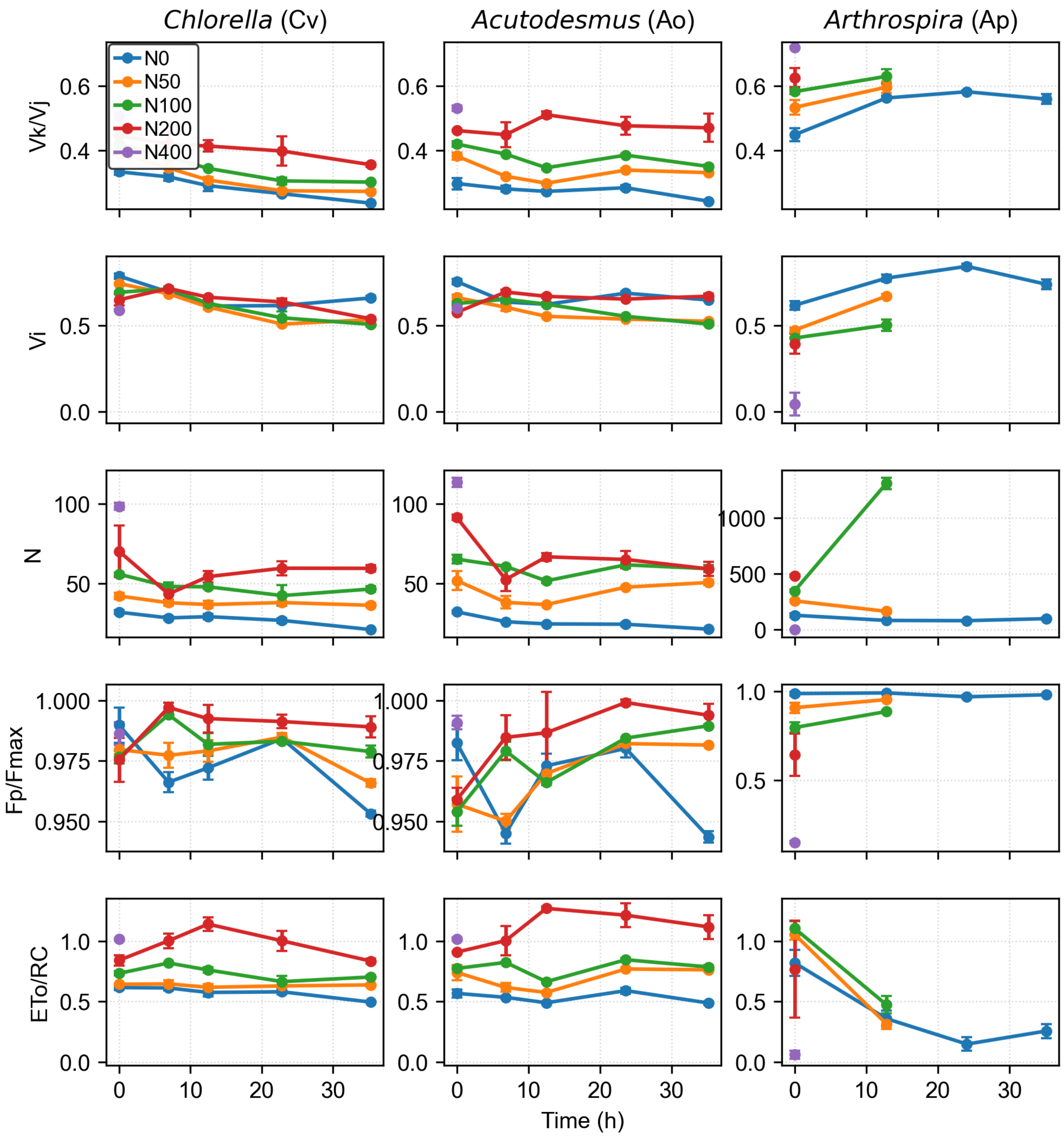


**Figure S3.** OJIP parameters over time (Culture 1) — Rows: Vk/Vj, Vi, N, Fp/Fmax, ETo/RC. Columns: species *Chlorella*, *Acutodesmus*, *Arthrospira*. Colors: $NH_3$ treatments (N0-N400). Error bars: mean ± standard deviation across replicates. Y-limits: for N and Fp/Fmax, *Chlorella* and *Acutodesmus* share limits; *Arthrospira* uses its own.

# 4. Correlation of Fv/Fm with $NH_3$ and specific growth rate

Scatter plots of Fv/Fm *vs* $NH_3$ concentration and *vs* specific growth rate (SGR). While Fv/Fm showed significant correlations, the variability increased when cultures experienced light inhibition, acclimation processes, or sustained toxicity. In particular, *A. obliquus* displayed relatively stable Fv/Fm, whereas *C. vulgaris* showed high variability at early time points, and *A. platensis* exhibited large scatter throughout the experiment. These findings indicate that Fv/Fm alone is not sufficient as a universal toxicity marker, especially under fluctuating outdoor cultivation conditions, underscoring the need for multivariate analysis.

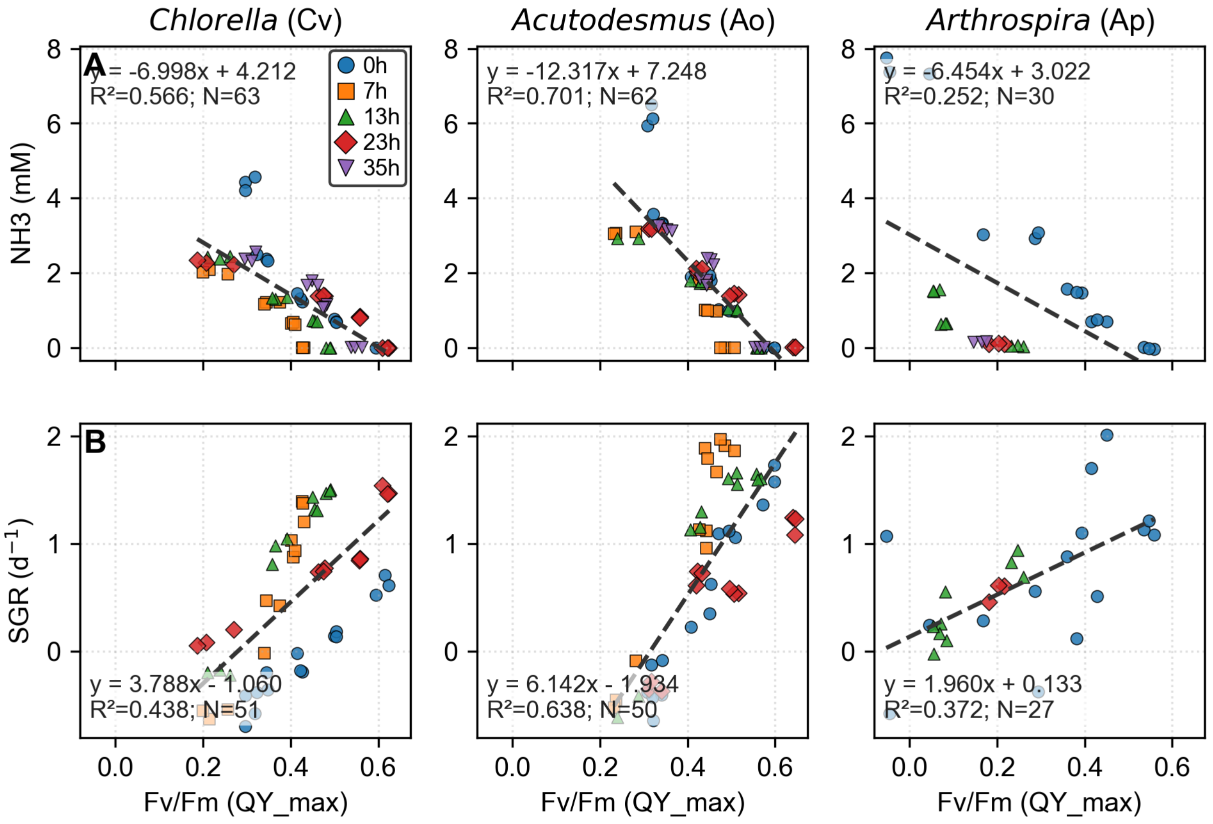


**Figure S4.** Correlations between Fv/Fm and (A) free ammonia ($NH_3$) concentration or (B) specific growth rate (SGR). Columns: *Chlorella* (Cv), *Acutodesmus* (Ao), *Arthrospira* (Ap). Markers/colors indicate sampling times. Lines show least-squares fits with regression equation and $R^2$.

# 5. $\alpha$ optimization of models

Cross-validation error curves across a logarithmic grid of α values for Lasso regression. As α increased, the number of selected variables decreased. This reduction sometimes increased variance but enhanced generalizability. The final α values were chosen using the 1-standard error (1-SE) rule, balancing accuracy and simplicity.

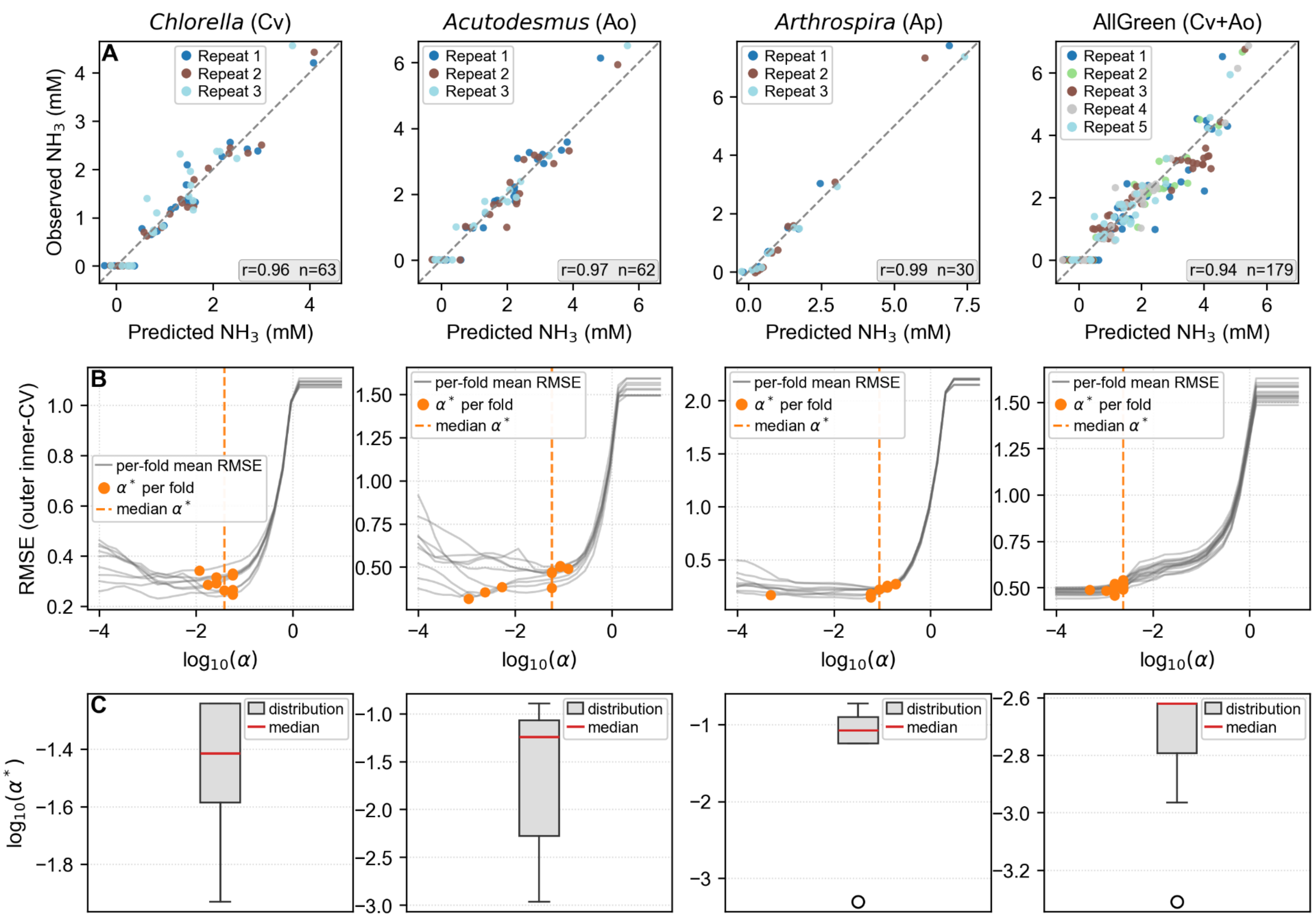


**Figure S5.** Alpha optimization across datasets Row 1: OOF parity (predicted vs observed $NH_3$) with dashed 1:1; inset shows Pearson r and sample size (n). Row 2: Inner-CV mean RMSE vs $\log_{10}(\alpha)$ per outer fold; orange markers at per-fold $\alpha^*$ (1SE), dashed line at median $\alpha^*$. Row 3: Distribution of $\alpha^*$ ($\log_{10}$ scale).

## 6. Model parameters in comparison of multivariable methods

When five multivariable methods, namely CCorA, Ridge, Lasso, Random Forest, and Gradient Boosting were compared, hyperparameters (parameters controlling model fitting) were optimized with grouped nested cross-validation (CV) (see Supporting Information section 8) with the OJIP data of *Chlorella*. The resulting optimized parameters were as follows.

Lasso: alpha = 0.0386
Ridge: alpha = 3.360
Random Forest:

- max_features: sqrt
- max_depth: 10
- n_estimators: 200

Gradient Boosting:

- max_depth: 2.0
- n_estimators: 200
- learning_rate: 0.05

## 7. Validation cultures (Culture 2)

Experimental overview of the validation culture (Culture 2), which involved higher inoculum density and shorter duration (14 h). $OD_{750}$ and Fv/Fm data are shown. Unlike in Culture 1, *A. platensis* maintained relatively high Fv/Fm values throughout the cultivation period in the control and low-$NH_3$ treatments, illustrating physiological variability between replicate cultures.

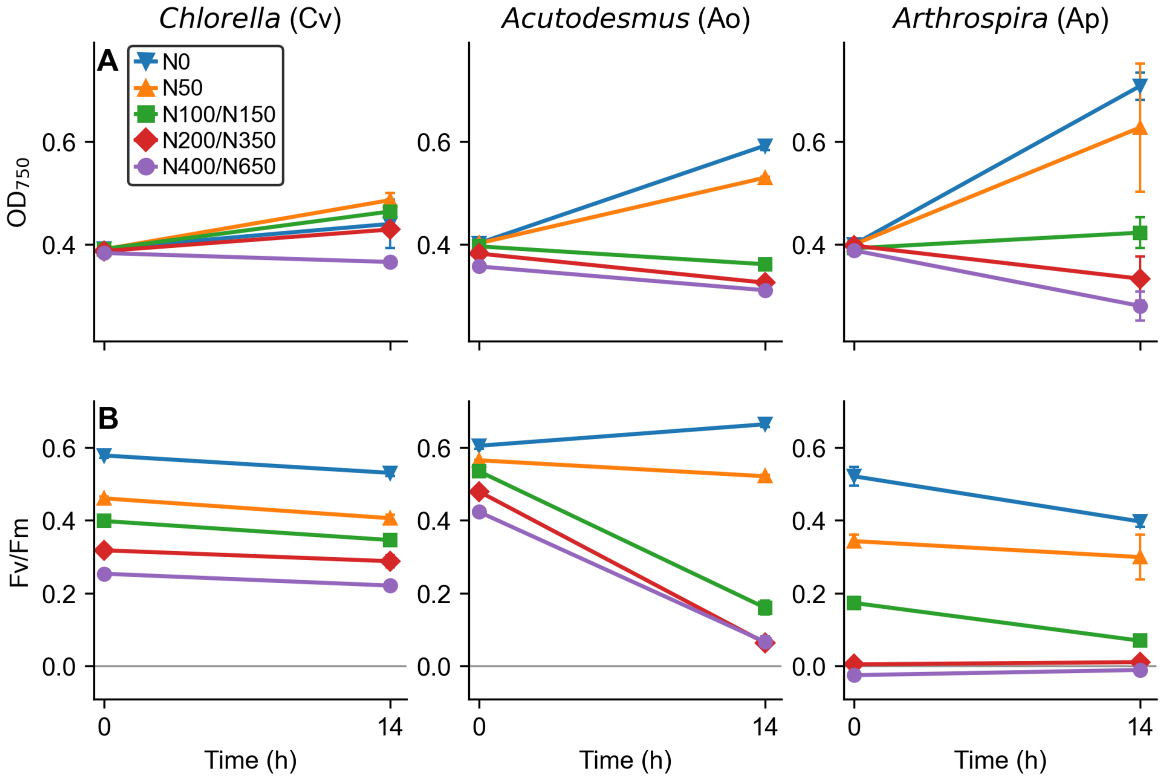


**Figure S6.** External validation culture (Culture 2) overview: (A) $OD_{750}$ and (B) Fv/Fm at 0 h and 14 h across $NH_3$ treatments. Colors and markers denote treatment tiers (Cv: N0–N400; Ao/Ap: N0–N650). Each column shows a species (Cv, Ao, Ap); points are mean±SD across replicates. $OD_{750}$ uses all replicates (no sample filters); Fv/Fm uses only the LowFo filter.

# 8. Model construction details

## a. Nested cross-validation (CV) and α optimization

Model optimization was conducted using a grouped nested cross-validation (CV) framework to obtain unbiased estimates of model generalization and to prevent information leakage (Krstajic et al., 2014; Varma & Simon, 2006; Varoquaux et al., 2017). All measurements originating from the same cultivation run or replicate were assigned to a single group, ensuring that near-identical samples never appeared in both training and test sets.

The outer loop consisted of a stratified group 5-fold CV, where groups were defined as *species* × *treatment* × *replicate*. One fold was held out for validation while the remaining folds were used for training. Within each outer-training split, an inner 5-fold group CV was conducted to tune the Lasso penalty parameter (α) across a log-spaced grid, following the one-standard-error (1-SE) rule (Hastie et al., 2009). All preprocessing steps, including feature scaling and target standardization, were fitted exclusively within the training data using a pipeline to avoid data leakage.

In Lasso regression, the coefficients $w$ were optimized by minimizing the objective function:

$$L(\alpha) = \frac{1}{2n} \parallel y - Xw \parallel^2 + \alpha \parallel w \parallel_1$$

where $L$ is the loss function (squared error plus L1 penalty), $X$ is the fluorescent feature matrix (n×p), $y$ is the target vector (n×1), $w$ is the coefficient vector (p×1), $n$ is the number of training samples, and $\alpha$ is the regularization strength.

The α value selected in the inner loop was fixed for refitting on each outer-training set and applied to predict its corresponding outer-validation fold. After completing all outer iterations, the median of the selected α values (α*) was used to refit a final species-specific model on the full training dataset (Culture 1).

Finally, a validation culture (Culture 2) collected under shifted experimental conditions (higher cell density and $NH_3$ concentration, shorter cultivation) served as a strict external test to evaluate the generalization of the model.

## b. Standardization of variables

All explanatory variables (fluorescence parameters) were standardized within each training fold by subtracting the fold-specific mean and dividing by the fold-specific standard deviation:

$$X' = \frac{X - \mu_{train}}{\sigma_{train}}$$

This ensured that the models were not biased by differences in scale among parameters. For prediction of new samples (e.g., Culture 2 validation data), the same mean ($\mu_{train}$) and standard deviation ($\sigma_{train}$) estimated from the training fold were applied, preventing information leakage.

The response variable ($NH_3$ concentration, Y) was also internally standardized during model training to improve comparability of α across folds:

$$Y' = \frac{Y - \mu_{Y,train}}{\sigma_{Y,train}}$$

This procedure stabilizes the effect of the Lasso penalty and ensures that α has consistent influence on coefficient shrinkage regardless of the scale of $NH_3$ concentrations. After prediction, standardized values ($Y'$) were back-transformed to the original $NH_3$ units using the mean and standard deviation from the corresponding training fold.

## c. Model building with optimized α

Final Lasso regression models were trained using the entire Culture 1 dataset, with both explanatory and response variables standardized as described above.

## d. Reliability measures (PI and AD)

- **Prediction Interval (PI):**

Prediction intervals were calibrated from the out-of-fold residuals obtained in the outer loop of nested cross-validation, following QSAR/QSPR validation practice to avoid optimistic bias (Krstajic et al., 2014; OECD, 2014; Tropsha et al., 2003). Let $r_i = y_i - \hat{y}_i$ be the residual for sample $i$ under its outer-fold prediction, and let $q_{0.975}(|r|)$ denote the 97.5$^{th}$ percentile of the empirical distribution of $|r_i|$. A two-sided 97.5% PI was computed as:

$$PI = \pm q_{0.975}(|r|)$$

This defines the expected error range for predictions. The interval for any prediction $\hat{y}$ is $[\hat{y} - PI, \hat{y} + PI]$.

- **Applicability Domain (AD):**

The applicability domain defines the multivariate space within which model predictions are considered reliable (OECD, 2014; Tropsha et al., 2003). In this study, the AD was quantified using a k-nearest neighbors (kNN) distance–based approach in the standardized feature space of fluorescence parameters (Jaworska et al., 2005; Sushko et al., 2010).

For each sample, the mean Euclidean distance to its $k$ nearest training neighbors was calculated in the active-feature space (i.e., features with non-zero Lasso coefficients). The number of neighbors was set adaptively as $k = \min(10, \max(3, \lfloor\sqrt{n_{\text{train}}}\rfloor))$ and then clipped to the valid range $1 \le k \le n_{train} - 1$, where $n_{train}$ is the number of training samples used to define the applicability domain. To align the AD metric with each feature's predictive contribution, the standardized features were additionally scaled by a coefficient-derived

weight $w_j = \sqrt{|\hat{\beta}_j| \,/\, \overline{(|\hat{\beta}|)}}$ , where $\hat{\beta}_j$ is the Lasso coefficient for feature $j$ and $\overline{|\hat{\beta}|}$ is the mean absolute coefficient across active features. This weighting amplifies distances along dimensions that strongly influence the prediction while attenuating the contribution of weakly informative features, thereby making the AD boundary model-specific rather than purely distributional. Samples whose weighted mean distance exceeded the 97.5th percentile of the training-set distance distribution were flagged as AD-out, indicating that their fluorescence features lie outside the region well represented by the model. This distance-based method provides a transparent and reproducible criterion to identify extrapolations beyond the trained data manifold.

### e. QC of validation data

Culture 2 datasets were processed using the same fluorescence QC criteria described in Section 1c.

### f. External validation

Predictions for Culture 2 samples were generated by applying the trained Lasso models, with input data standardized using the parameters from Culture 1 training. Results were classified as AD-in/out and PI-in/out, and predictive performance was summarized accordingly.

## 9. Inclusion of Light-Curve data in model training and prediction

Incorporation of data from light curve protocol (LC2 on AquaPen) on top of OJIP data for training of Lasso prediction models was attempted. As a result, prediction accuracy did not improve very much (Fig. S7; in comparison with Fig. 5 in the main text). In *Chlorella* and *Acutodesmus* models, PI-out values decreased, but this was most likely owing to slightly wider PI values (see Fig. 4 in the main text for comparison). In conclusion, no apparent improvement was observed through incorporation of LC2 data.

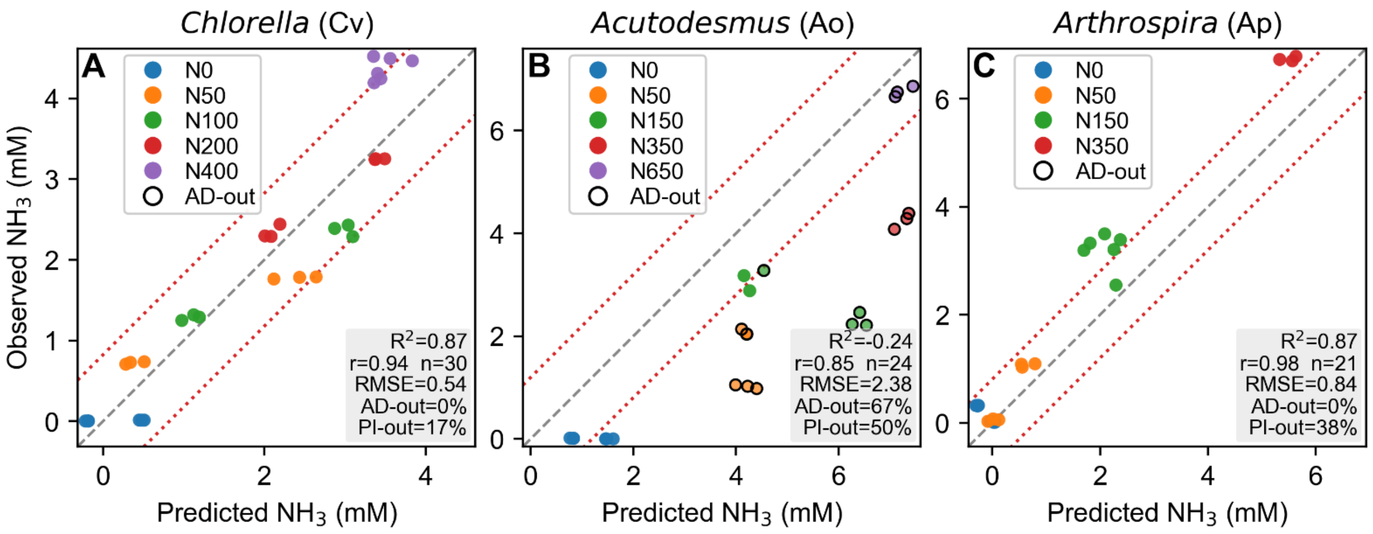


**Figure S7.** External validation of models trained with OJIP & light-curve (LC2) parameters. Prediction intervals (PI) for each species were ±0.82 (*Chlorella*), ±1.20 (*Acutodesmus*), and +±0.80 (*Arthrospira*), respectively.

## 10. Time-course pH variation

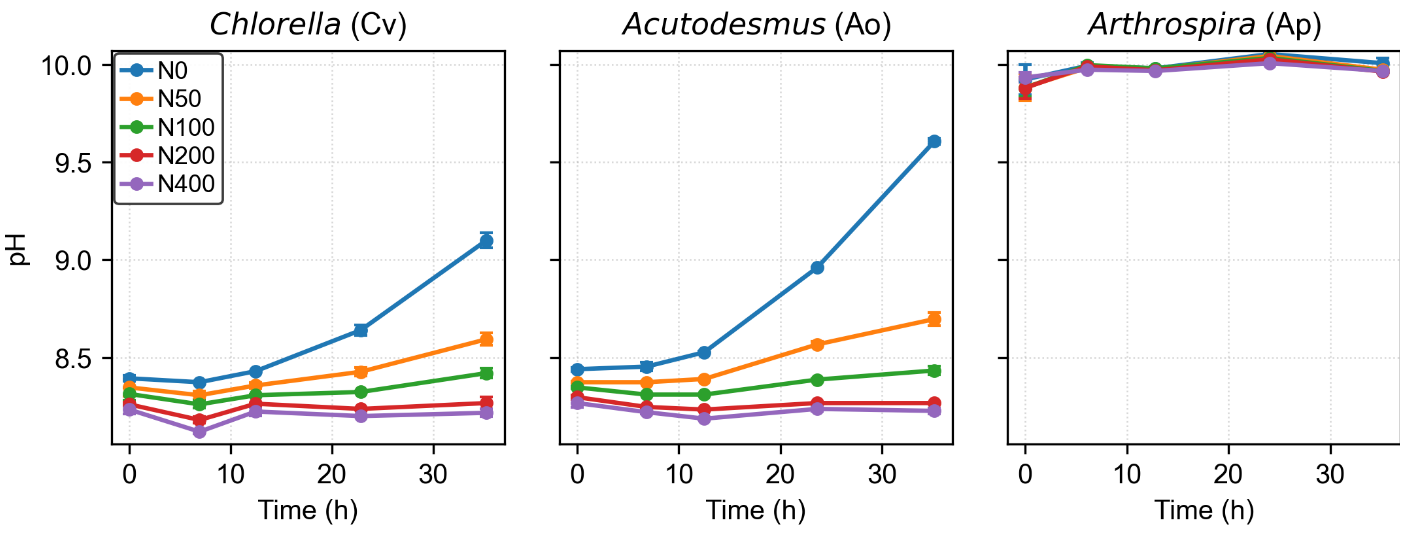


**Figure S8.** pH time-course in Culture 1. Columns: *Chlorella* (Cv), *Acutodesmus* (Ao), *Arthrospira* (Ap). Colors: $NH_3$ treatments (N0-N400). Error bars: mean ± standard deviation across replicates.

**References**


Jaworska, J., Nikolova-Jeliazkova, N., & Aldenberg, T. (2005). QSAR Applicability Domain Estimation by Projection of the Training Set in Descriptor Space: A Review. *Alternatives to Laboratory Animals*, *33*(5), 445–459. https://doi.org/10.1177/026119290503300508

Kalaji, H. M., Jajoo, A., Oukarroum, A., Brestic, M., Zivcak, M., Samborska, I. A., Cetner, M. D., Łukasik, I., Goltsev, V., & Ladle, R. J. (2016). Chlorophyll a fluorescence as a tool to monitor physiological status of plants under abiotic stress conditions. *Acta Physiologiae Plantarum*, *38*(4), 102. https://doi.org/10.1007/s11738-016-2113-y

Krstajic, D., Buturovic, L. J., Leahy, D. E., & Thomas, S. (2014). Cross-validation pitfalls when selecting and assessing regression and classification models. *Journal of Cheminformatics*, *6*(1), 1–15. https://doi.org/10.1186/1758-2946-6-10

OECD. (2014). *Guidance Document on the Validation of (Quantitative) Structure-Activity Relationship [(Q)SAR] Models* (OECD Series on Testing and Assessment). OECD. https://doi.org/10.1787/9789264085442-EN

Stirbet, A., & Govindjee. (2011). On the relation between the Kautsky effect (chlorophyll a fluorescence induction) and Photosystem II: Basics and applications of the OJIP fluorescence transient. *Journal of Photochemistry and Photobiology B: Biology*, *104*(1–2), 236–257. https://doi.org/10.1016/j.jphotobiol.2010.12.010

Strasser, R. J., Srivastava, A., & Tsimilli-Michael, M. (2000). The fluorescence transient as a tool to characterize and screen photosynthetic samples. In *Probing Photosynthesis: Mechanism, Regulation & Adaptation* (Issue January, pp. 443–480).

Sushko, I., Novotarskyi, S., Körner, R., Pandey, A. K., Cherkasov, A., Li, J., Gramatica, P., Hansen, K., Schroeter, T., Müller, K. R., Xi, L., Liu, H., Yao, X., Öberg, T., Hormozdiari, F., Dao, P., Sahinalp, C.,

Todeschini, R., Polishchuk, P., … Tetko, I. V. (2010). Applicability Domains for Classification Problems: Benchmarking of Distance to Models for Ames Mutagenicity Set. *Journal of Chemical Information and Modeling*, *50*(12), 2094–2111. https://doi.org/10.1021/CI100253R

Tropsha, A., Gramatica, P., & Gombar, V. K. (2003). The importance of being earnest: Validation is the absolute essential for successful application and interpretation of QSPR models. *QSAR and Combinatorial Science*, *22*(1), 69–77. https://doi.org/10.1002/qsar.200390007

Varma, S., & Simon, R. (2006). Bias in error estimation when using cross-validation for model selection. *BMC Bioinformatics*, *7*(1), 1–8. https://doi.org/10.1186/1471-2105-7-91

Varoquaux, G., Raamana, P. R., Engemann, D. A., Hoyos-Idrobo, A., Schwartz, Y., & Thirion, B. (2017). Assessing and tuning brain decoders: Cross-validation, caveats, and guidelines. *NeuroImage*, *145*, 166–179. https://doi.org/10.1016/J.NEUROIMAGE.2016.10.038
